# Metabolic Syndrome and Biotherapeutic Activity of Dairy (Cow and Buffalo) Milk Proteins and Peptides: Fast Food-Induced Obesity Perspective—A Narrative Review

**DOI:** 10.3390/biom14040478

**Published:** 2024-04-14

**Authors:** Kenbon Beyene Abdisa, Emőke Szerdahelyi, Máté András Molnár, László Friedrich, Zoltán Lakner, András Koris, Attila Toth, Arijit Nath

**Affiliations:** 1 Department of Food Process Engineering, Institute of Food Science and Technology, Hungarian University of Agriculture and Life Sciences, Ménesi út 44, HU-1118 Budapest, Hungary; kenbonb@gmail.com (K.B.A.);; 2 Department of Nutrition, Institute of Food Science and Technology, Hungarian University of Agriculture and Life Sciences, Somlói út 14-16, HU-1118 Budapest, Hungary; nemethne.szerdahelyi.emoke@uni-mate.hu; 3 Department of Refrigeration and Livestock Product Technology, Institute of Food Science and Technology, Hungarian University of Agriculture and Life Sciences, Ménesi út 43-45, HU-1118 Budapest, Hungary; 4 Department of Agricultural Business and Economics, Institute of Agricultural and Food Economics, Hungarian University of Agriculture and Life Sciences, Villányi út 29-43, HU-1118 Budapest, Hungary; 5 Division of Clinical Physiology, Department of Cardiology, Faculty of Medicine, University of Debrecen, Móricz Zsigmond út 22, HU-4032 Debrecen, Hungary

**Keywords:** metabolic syndromes, metabolic factors, hyperglycemia, dyslipidemia, fast food-induced obesity, dairy milk protein, peptides

## Abstract

Metabolic syndrome (MS) is defined by the outcome of interconnected metabolic factors that directly increase the prevalence of obesity and other metabolic diseases. Currently, obesity is considered one of the most relevant topics of discussion because an epidemic heave of the incidence of obesity in both developing and underdeveloped countries has been reached. According to the World Obesity Atlas 2023 report, 38% of the world population are presently either obese or overweight. One of the causes of obesity is an imbalance of energy intake and energy expenditure, where nutritional imbalance due to consumption of high-calorie fast foods play a pivotal role. The dynamic interactions among different risk factors of obesity are highly complex; however, the underpinnings of hyperglycemia and dyslipidemia for obesity incidence are recognized. Fast foods, primarily composed of soluble carbohydrates, non-nutritive artificial sweeteners, saturated fats, and complexes of macronutrients (protein-carbohydrate, starch-lipid, starch-lipid-protein) provide high metabolic calories. Several experimental studies have pointed out that dairy proteins and peptides may modulate the activities of risk factors of obesity. To justify the results precisely, peptides from dairy milk proteins were synthesized under in vitro conditions and their contributions to biomarkers of obesity were assessed. Comprehensive information about the impact of proteins and peptides from dairy milks on fast food-induced obesity is presented in this narrative review article.

## 1. Introduction

**Figure 1:**
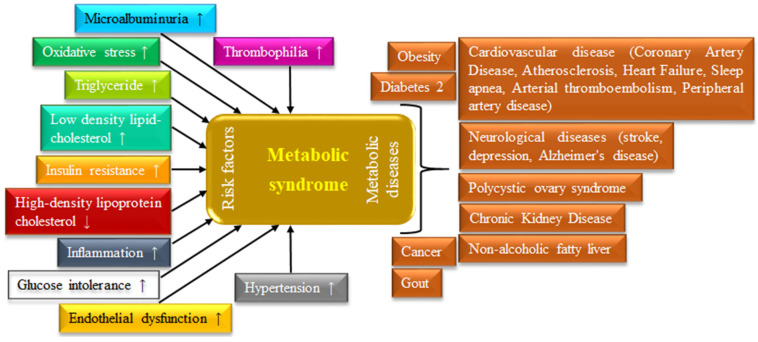
Risk factors of MS and associated metabolic diseases (self-developed, concept was adopted from Mendrick et al., 2018 [7]).

Metabolic syndrome (MS) is a consequence of the interplay between different metabolic factors that directly increase the risks of a wide range of diseases. Over the last decade, it has been considered one of the leading causes of morbidity and mortality [1], including premature death [2]. According to the medical classification list of the World Health Organization (WHO), MS has been recognized as the International Statistical Classification of Diseases and Related Health Problems (ICD) with ICD-9 and ICD-10 having codes 277.7 and E88.81, respectively [3]. There are 16 different possible combinations of risk factors that could be diagnosed for MS; however, not all can be considered equally in terms of their impact on risk. It may be supposed that interconnections of some metabolic risk factors, such as insulin resistance, hypertension, glucose intolerance, oxidative stress, inflammation, dyslipidemia (elevated triglycerides (TGs) and low-density lipid-cholesterol (LDL-C), and lower levels of high-density lipid-cholesterol (HDL-C)), thrombophilia, microalbuminuria, and endothelial dysfunction may be considered influencing factors for various diseases, such as diabetes mellitus (DM), cancer, non-alcoholic fatty liver disease (NAFLD), neurological diseases (stroke, depression, and Alzheimer’s disease), polycystic ovary syndrome, chronic kidney disease, nonalcoholic steatohepatitis, gout, cardiovascular disease (coronary artery disease, atherosclerosis, heart failure, sleep apnea, arterial thromboembolism, peripheral artery disease), and many more [4]. Thus, MS may certainly be viewed as a risk syndrome, and it is well recognized that patients with MS are more frequently hospitalized and have greater drug expenditure [5]. MS is characterized by some major signs, such as a rise in visceral obesity, dyslipidemia, hyperglycemia, and elevated blood pressure and hypertension. Another characteristic of MS is chronic low-grade inflammation as a consequence of the complex interplay between different risk factors [6]. Different risk factors for MS and associated diseases are presented in Figure 1.

The concept of MS as the association of hypertension, hyperglycemia and gout was demonstrated by Swedish physician Kylin in 1920 [4]. Vague in 1947 described that visceral obesity is usually associated with the metabolic abnormalities found in DM and cardiovascular diseases (CVDs) [8]. Avogaro and Crepaldi described a syndrome which comprised hypertension, hyperglycemia, and obesity at the European Association for the Study of Diabetes annual meeting in 1965 [9]. The term “metabolic syndrome” was first used by Haller and Hanefeld in 1975. They conferred that MS is a combination of simultaneous risk factors of CVDs and diabetes that occur together more frequently than by chance alone [10]. In 1988, Reaven mentioned “a cluster of risk factors for diabetes and cardiovascular disease” and named it “Syndrome X”, and insulin resistance as the “driving force” of the syndrome was proposed [11]. Subsequently in 1989, Kaplan renamed the syndrome “The Deadly Quartet” for the combination of upper body obesity, glucose intolerance, hypertriglyceridemia, and hypertension [12]. It was again renamed “The Insulin Resistance Syndrome” in 1992 [13]. Several diagnostic criteria, such as insulin resistance, body weight, blood pressure, glucose and lipid were reported to define MS. The most commonly used criteria for defining MS are from the WHO [14], the International Diabetes Federation (IDF) [15], the European Group for the study of Insulin Resistance (EGIR) [16] and the American Association of Clinical Endocrinologists (AACE) [17]. Diagnostic criteria proposed for defining MS by different authorized bodies are presented in Table 1.

However, each definition holds some common features, there are several parameters that differ according to authorized bodies. Different diagnostic criteria partly influence the estimation of the prevalence of MS. Therefore, it may be realized that accurate estimation of the prevalence of MS is quite difficult. The global prevalence of MS depends on several issues, such as region, urban or rural environment, composition of the population (sex, age, race and ethnicity), no matter which criteria are used to estimate the prevalence of MS [18]. In addition to genetic and epigenetic factors, it was proven that physical inactivity, environmental contaminants, an unhealthy lifestyle and diet are significantly associated with a wide range of metabolic diseases [19]. Therefore, MS comprises several complex biochemical pathways and mechanisms that are not fully comprehended. It has been estimated that the prevalence of MS ranges from around 10% to 30% of the world’s adult population, depending on the diagnostic criteria suggested by the Adult Treatment Panel III of the National Cholesterol Education Program (ATP-III) and the Joint Interim Statement (JIS) [20]. There have been several studies on the medical costs of MS and related diseases because the economic burden of MS has been greatly increasing [21,22].

Presently, technological advances have enabled more precise, intrinsic, high-throughput analysis by different types of omics results. Multi-omics concepts, including genetics, transcriptomics, epigenetics, proteomics and metabolomics have been considered as a promising approach to detect, characterize and understand the pathophysiology of different metabolic diseases [23]. However, several risk factors of MS were identified; the pipelines between them are complicated, still unclear and often contrasting results were published [24]. It may be believed that MS is primarily contributed to by overproduction of reactive oxygen species (ROSs), inflammatory biomarkers, dysfunctional adipocytes (accumulation in visceral fat), and insulin resistance [25,26] and prothrombotic state [27]. Furthermore, an unhealthy gut microbiome and environmental contaminants in the food chain modulate risk factors of MS [7]. Several meta-analyses of experimental evidence indicated that the environmental contaminant Dichlorodiphenyltrichloroethane (DDT) and its derivatives, such as Dichlorodiphenyldichloroe-thylene (DDE) in the food chain, modulate mitochondrial dynamics (mitochondrial fusion and fission, mitophagy and mitochondrial biogenesis), mitochondrial function and the prevalence of insulin resistance [28]. Results of metagenomic studies of fecal microbiota and association of circulating trimethylamine N-oxide (TMAO) in pathological conditions reveal the contribution of gut microbiota in the prevalence of MS [29,30].

**Figure 2:**
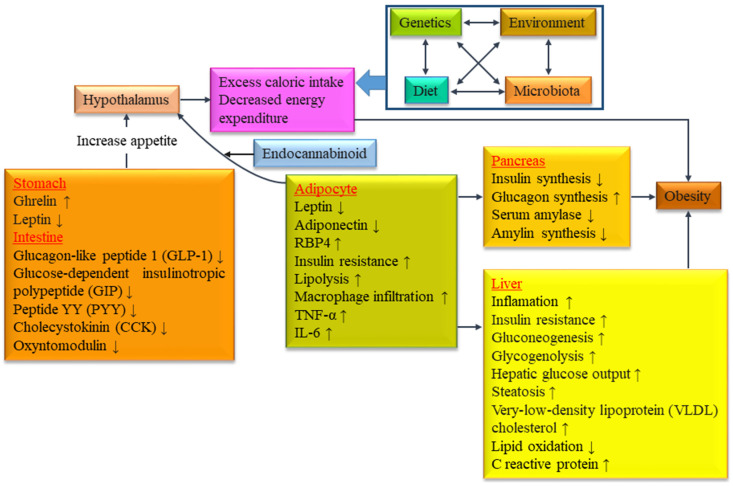
Interconnection between genetic factors, environmental factors, dietary pattern and gut microbiota associated with the incidence of obesity (self-developed, concept was adopted from Ussar et al., 2016 [36]).

According to the WHO, IDF, EGIR and AACE, the impacts of two major metabolic diseases, such as dyslipidemia and hyperglycemia, on mortality, morbidity and therapeutic expenditures are rising [10]. Obesity is a multifactorial disorder, influenced by genetic factors, dietary pattern, gut microbiota and environmental factors. Mutations in various genes responsible for controlling appetite and metabolism are primary causes of obesity; however, several investigations documented that lifestyle factors including dietary patterns have contributed to an increased occurrence of hyperglycemia and dyslipidemia, resulting in an increased prevalence of obesity [31,32]. Furthermore, gene-environment interactions play a significant role in MS including obesity. According to epidemiologic studies in 2016, it was estimated that obesity can affect 1.12 billion persons by 2030 [33]. The recent Coronavirus disease 2019 (COVID-19) pandemic also influenced illness severity and obesity. It has been recognized that hyperglycemia in the non-diabetic range is an important risk factor for COVID-19 [34]. However, the underpinnings of the pathogenesis of obesity are not yet fully understood, it was confirmed that the etiology of obesity is the imbalance between energy intake and expenditure, which is related with dietary intake (nutritional imbalance due to mainly empty calorie food intake) and lifestyle [35]. Complex multidirectional interactions, described by the “Interaction Model” in the case of obesity, are presented in Figure 2.

Presently, fast foods are being taken into consideration in dietary patterns due to the rapid socio-economic transition, taste preference, the relatively low cost for large portion sizes and quick availability [37]. It has been proven by several investigations that consumption of fast foods is linked with the mentioned two metabolic diseases, which may lead to an increased risk of obesity and a number of non-communicable diseases [38,39]. Typically, high-calorie fast foods are composed with of amounts of soluble carbohydrates, non-nutritive artificial sweeteners, saturated fats, and protein-carbohydrate (Maillard products), starch-lipid and starch-lipid-protein complexes [40]. The nutritional values of foods are lost due to the biochemical modulation of macro- and micro- nutrients during cooking and processing at high temperature [41]. Deep frying, spray drying, microwave heating and extrusion processes are widely used for the preparation of a wide range of fast foods. The oil-food interaction at high temperatures due to the deep frying process leads to physical and chemical changes, such as starch gelatinization, protein denaturation, flavor and color formation, and many more [42]. Edible oils undergo various biochemical modifications during frying at high temperature and massive amounts of toxic active compounds are produced. These products are generally nonvolatile (epoxides, oxidized polymers, carbonyls, and polar dimers) and volatile (aldehydes, alcohols, and hydrocarbons). These toxic compounds are considered potential carcinogenic, mutagenic, genotoxic and teratogenic substances for human health [43]. Due to prolonged heating of cooking oils, TGs begin to break down to free fatty acids (FFAs) and glycerol, and glycerol is quickly dehydrated to acrolein. A wide range of potentially toxic compounds in the class of aldehydes and polycyclic aromatic hydrocarbons were identified in deep fried foods [44]. The lipid peroxidation of fatty acids leads to the formation of lipid peroxides, which first convert to reactive dicarbonyls and finally to advanced glycation end products (AGEs) [45]. Starch-lipid and starch-lipid-protein complexes are produced by a series of noncovalent interactions, including hydrogen bonds, hydrophobic attractions and van der Waals forces among macronutrients during the deep frying process [46]. One common complex biomolecule in fast foods is Maillard products. They are produced by a Maillard reaction, where a covalent bond forms between a free amino group in protein and a carbonyl group of a reducing carbohydrate of polysaccharide due to cooking or processing at high temperature. A series of reactions, such as the formation of a Schiff base, followed by an Amadori rearrangement and subsequently oxidative modifications (glycoxidation) are involved to produce Maillard products [47]. However, Maillard products are produced as a part of the healthy metabolism of carbohydrates, but consumption of excessively high amounts of Maillard products is not appreciable. Apart from Maillard products, several harmful substances and toxic compounds, such as furan, heterocyclic aromatic amines, acrylamide, acrolein and trans fatty acids were identified in food matrices produced by high temperature. They have the ability to promote oxidative stress and inflammation by binding with cell surface receptors or cross-linking with intracellular proteins, and modulate their structure and function [47]. Furthermore, the use of soluble carbohydrates and non-nutritive high-intensity sweeteners, such as aspartame, neotame, acesulfame potassium, advantame, saccharin and sucralose in fast foods has been reported [48]. Non-nutritive sweeteners modulate the secretions of insulin and anorexigenic (appetite suppressing) hormones (glucagon-like peptide 1 (GLP-1), peptide YY (PYY) and cholecystokinin (CCK)), insulin resistance, composition of gut microbiota and postbiotics, mainly short-chain fatty acids (SCFAs) [48].

Obesity is a heterogeneous and multifactorial health disorder; however, it is a well-established fact that the risk of obesity is associated with food intake, appetite and energy balance. Currently, obesity due to consumption of fast foods is considered one of the most relevant topics of discussion in the context of the management of MS because an epidemic heave of the incidence of obesity in both developing and underdeveloped countries has been reached. Furthermore, it has been proven that moderate and higher affinity toward a Western diet is associated with a higher risk of obesity and COVID-19 infection during the pandemic [49]. The primary objective of this article is to make a link between two major metabolic diseases, such as hyperglycemia and dyslipidemia with fast food-induced obesity. Another objective of this review is to highlight the beneficial role of dairy milk (cow and buffalo) proteins and peptides in dietary choice to combat obesity prevalence. Therefore, inclusive information about obesity contributed to by hyperglycemia and dyslipidemia due to consumption of high-calorie fast foods is presented in this narrative review article. Subsequently, the therapeutics and beneficial attributes of dairy milk-based proteins and peptides for fast food-induced obesity are mentioned with a biochemical viewpoint and up-to-date results of clinical trials in this narrative review article.

## 2. Methodology

A rapid scoping review was conducted. It was conducted to map evidence on the mentioned topic, ascertain information from the available literature, summarize information and concepts, identify prime concepts and theories, and identify the gaps in the existing knowledge with the aid of planning and commissioning future research. The present scoping review fits with the questions of aims and objectives which sit across diverse research. It allowed for a broad search and the review was reported following the PRISMA Scoping Reviews (PRISMA-ScR) Checklist [50].

## 3. Obesity

Obesity has been recognized as a disease since 1948 by the WHO and in 1997, the WHO recognized this complex and chronic disease is not just constrained to the affluent in Western nations. According to the International Classification of Diseases 11 (ICD-11), obesity is a chronic complex and multifactorial disorder, characterized by an excess of body fat. Obesity is associated with higher consumption of carbohydrates and fat, and a wide range of metabolic dysfunctions, which affect multiple organs of the body and subsequently disrupt their regular function. The epidemic heave of obesity has spread beyond the borders of high-income nations to low- and middle-income countries, predominantly in urban areas [51]. Presently, probable different biomarkers and molecular mechanisms of Western diet-induced obesity and obesity-related carcinogenesis were published. The contribution of insulin resistance, hyperglycemia, hypertriglyceridemia, hypoxia, oxidative stress, mitochondrial dysfunction, dysregulation of glycolysis and lipogenesis, adipokine/cytokine/exosome release, angiogenesis and epithelial to mesenchymal transition (EMT) have been recognized [52].

The prevalence of MS is increasing among adults worldwide and it is largely related to possible factors, such as heredity, epigenetic modifications, socioeconomic status, diets, gut dysbiosis, sedentary lifestyle, medication, medical condition and high body mass index (BMI) [53]. According to the WHO in 2019, more than 1 billion people in the world were obese, including 650 million adults, 340 million adolescents and 39 million children [54]. In another report, there were ~2 billion adults who were overweight, while 650 million were obese. It may be realized that if this rate will not slow down, 2.7 billion adults might be overweight and over 1 billion will be obese by 2025 [55]. According to the World Obesity Atlas 2023 report, 38% of the world population are currently either overweight or obese [56].

It is proven that the central nervous system plays an essential role in the control of food intake and energy homeostasis through the expressions of a wide range of genes in the hypothalamus and pituitary gland. In the early phase of the 20th century, it was assumed that dysfunction of the pituitary/hypothalamus leads to obesity. During the 1940s to 1970s, it was assumed that psychological and psychodynamic aspects are the key factors leading to obesity. In 1956, obesity was introduced as a syndrome. The role of genetic factors in body weight regulation came to the forefront in the 1980s and early 1990s [57]. Recently, it is stated that two factors, such as (a) obesogenic environment triggers obesity-predisposing genes and (b) epigenetic modulation contribute to obesity incidence. The first genome-wide association studies (GWAS) for obesity traits were published in 2007. The first identified obesity-susceptibility locus was the *FTO* gene (fat mass obesity associated) and until now this gene is recognized as the highest contributor to the risk of obesity [58]. Subsequently, many more GWAS were considered and approximately 60 GWAS identified more than 1100 independent loci associated with a range of obesity outcomes [59]. Genomic studies reveal that more than 300 single-nucleotide polymorphisms and 227 genetic variants are related to obesity, although their functional attributions on the obese phenotype are still unclear [60]. Genetic variations, such as single-nucleotide polymorphisms (SNPs), copy number variants (CNVs), and small insertions and deletions of nucleotide sequences contribute to the development of obesity [61,62]. Obesity can be classified into three categories based on genetic contribution. Those are: (a) common polygenic obesity, outcome of the interaction between environmental factors and genetic susceptibilities, (b) syndromic obesity, characterized by obesity combined with developmental delay or dysmorphism and (c) monogenic obesity, outcome of pathogenic variants in single genes, generally involved in the hypothalamic leptin-melanocortin pathway along with the regulation of satiety and hunger [63]. Common polygenic obesity has high prevalence, does not follow the principles of Mendelian inheritance and heritability similar to other multifactorial diseases. In this case, a wide range of variants in several genes interact with environmental factors. Monogenic obesity has a low prevalence, follows a Mendelian inheritance pattern and early onset devastated clinical manifestations. In the case of monogenic obesity, genetic variants in single genes lead to large effects [62]. Furthermore, obesity can be classified into the following categories, such as overweight (BMI = 25 to < 30 kg·m^−2^), moderate obesity (BMI = 30 to <35 kg·m^−2^) and severe obesity (BMI ≥ 35 kg·m^−2^) [64]. Contributions of leptin, leptin receptor, proopiomelanocortin, prohormone convertase 1, melanocortin 4 receptor, single-minded homolog 1, brain-derived neurotrophic factor and neurotrophic tyrosine kinase receptor type 2 genes have been identified as causes for obesity [65,66]; however, more than 500 obesity-related genes were identified [67]. High maternal BMI, high gestational weight gain and diabetes may cause metabolic disorders including obesity in offspring. The epigenetic modifications and imbalance of unhealthy nutritional supply to the fetus in the maternal phase may influence the development of the fetus, fat accumulation and other metabolic complications [68]. Obesity experiences in infancy and childhood phase may predispose to adult obesity. Furthermore, it has been proven that children with obesity are five times more likely to be obese in adulthood [69].

It has been proven that obesity plays an important role in the homeostatic regulation of systemic glucose. Patients with type 1 DM (T1DM) are hardly obese at the time of assessment and diagnosis; whereas patients can be overweight or obese due to insulin resistance and impaired glucose tolerance. Subsequently, it manifests a hyperglycemic condition, characteristic of T2DM. Due to high body fat content, patients with T2DM often experience various types of cardiovascular risk factors, such as hypertension and dyslipidemia (low high-density lipoprotein and/or elevated TGs) [70,71]. On the other hand, a woman may experience hypertension and dyslipidemia prior to GDM [66]. Apart from diabetes and dyslipidemia, obesity is correlated with other metabolic disorders, including NAFLD, CVDs, chronic kidney diseases and cancers [72]. Therefore, the effects of obesity attribute a tremendous financial burden on the health care system. Several investigations indicate that medical costs of obesity management were higher compared to individuals with normal body weight [73]. Obesity is characterized by changes in metabolic pathways and complex metabolic imbalance related to energy balance, glucose, lipid and adipose tissue homeostasis, dysregulation of other physiological processes, including the activity of the central and peripheral nervous system, and their interactions.

### 3.1. Diabetes

DM has a long history reaching back to antiquity, dated back to 1500 BC. It is a chronic metabolic disease involving all types of macronutrients (carbohydrate, protein and lipid) metabolism. DM is characterized by a physiologically abnormal hyperglycemic condition, represented by continued high levels of blood glucose as a result of a wide range of biochemical pathways, predominantly related to insulin secretion or its action or both. Diabetes with its ever-increasing global prevalence is parallel to the rapid economic growth, urbanization and adoption of modern lifestyle [74]. In the year 2017, diabetes had a global 22.9 million incidences, 476.0 million prevalence, 1.37 million deaths and 67.9 million disability-adjusted life-years. It was projected that in 2025, diabetes may cause 26.6 million incidences, 570.9 million prevalence, 1.59 million deaths and 79.3 million disability-adjusted life-years [75].

Diabetes may be classified into four categories based on both etiology and pathogenesis of the disease. Those are T1DM (accounts for 5–10% of all diabetic cases), T2DM (accounts for 90–95% of all diabetic cases), gestational DM (GDM) (1–14% of all pregnancies) and secondary diabetes caused or associated with certain specific conditions, pathologies, and/or disorders (constitute a smaller percentage) [70,71]. T1DM, also known as insulin-dependent DM (IDDM) or juvenile-onset diabetes, is an autoimmune disorder characterized by T-cell-mediated destruction of β-cells in the pancreas, which results in insulin deficiency and later hyperglycemia [76]. T2DM or adult-onset diabetes is non-insulin-dependent DM (NIDDM) and is characterized by two main insulin-related irregularities, such as insulin resistance and insulin deficiency due to the dysfunction of pancreatic β-cells [77]. GDM is defined as higher fasting and post-prandial blood glucose levels or any degree of glucose intolerance at the onset of or during pregnancy, generally in the second or third trimester [78]. Furthermore, T1DM, T2DM and GDM, secondary diabetes may appear due to some specific conditions including various pathologies and/or several disorders. The majority among this type of diabetes includes maturity-onset diabetes of the young (MODY), neonatal diabetes, lipoatrophic diabetes, drug or chemical-induced diabetes, ketosis-prone diabetes mellitus, and so on [79]. Hyperglycemia and its associated metabolic pathways affect multiple organs of the body and disrupt their normal function, which leads to micro- and macrovascular complications. These complications are manifested in a wide range of body organs and systems, such as the kidney, heart, skin and nerves [70].

One of the major factors responsible for T2DM is the dysfunction of pancreatic β-cells. As an outcome of the dysfunction of pancreatic β-cells and consequently inadequate secretion of insulin, postprandial and subsequently fasting glucose levels are increased. It reduces glucose production in hepatocytes and increases glucose uptake in muscle and hepatocytes. In early diabetic conditions, complications appear to be insufficient responsiveness of pancreatic β-cells to glucose and later it turns to the reduction in pancreatic β-cell mass. The elevated plasma glucose in the case of diabetes might subsidize further disease advancement through glucotoxic effects on pancreatic β-cells and insulin sensitivity [80]. However, the classic pathogenesis of T2DM was considered an insulin deficiency from pancreatic β-cells only, later the contribution of insulin resistance was introduced. Insulin resistance is clinically defined as the incapability of a known amount of insulin (endogenous or exogenous) to escalate glucose uptake and utilization in an individual similar to a usual one [26]. A number of genes are associated with the dysfunction of pancreatic β-cells and insulin resistance in T2DM. Among those genes, the transcription factor TCF7L2 is well studied [81,82]. Glucose homeostasis is maintained by the activation of the transcription factor *FOXO1* in the liver and impaired skeletal muscle GLUT4 translocation. *FOXO1* induces the activities of key enzymes of gluconeogenesis; hence, its upregulation increases the formation of glucose in hepatocytes. In hepatocytes, insulin generally involves phosphorylation and suppression of *FOXO1* activity through the action of protein kinase Akt [83]. Downregulation of GLUT4 translocation to the plasma membrane is the cause of insulin resistance. Interestingly, it has been reported that though GLUT4 level is reduced by 50% in adipose tissue in the case of T2DM, it is unchanged in skeletal muscle [84]. However, non-esterified fatty acids (NEFAs) are critical for normal insulin release, high levels of the formation of NEFA by a lipid infusion-induced pancreatic β-cell dysfunction and insulin resistance in T2DM. The lipotoxicity due to the formation of NEFA in diabetic individuals may cause more deleterious effects, known as ‘glucolipotoxicity’ [85]. Furthermore, the declined capability of adipocytes to preserve and retain TG in obese individuals, resulting in the accumulation of ectopic fat in hepatocytes and muscle cells, which may cause insulin resistance. Signaling of insulin through Akt in hepatocytes activates fatty acid biosynthesis from glucose and amino acids (de novo lipogenesis (DNL)), which extended the conversion of TG to very-low-density lipoprotein (VLDL) and subsequently, accumulated in peripheral tissues [86,87]. Insulin sensitivity modulates the activity of pancreatic β-cells and it is decreased in the case of obesity. Dysfunction of pancreatic β-cells is noted with increasing fasting glucose level. It is important to mention that fasting hyperinsulinemia without detectable elevations in blood-glucose concentrations is generally noted in some individuals with long-established obesity [88]. Insulin resistance is manifested in both the hepatic and peripheral tissues. Hepatic insulin resistance results in over accumulation of glucose in the basal state, despite the presence of fasting hyperinsulinemia and hyperglycemia. Insulin resistance of peripheral tissues, mainly muscle, reduces insulin-stimulated glucose uptake and postprandial hyperglycemia [89]. Chronic elevated glucose level leads to the generation of ROSs which increase oxidative stress in a wide range of pancreatic cells followed by the dysfunction of pancreatic activity [90]. As a consequence, secretions of insulin and amylin from pancreatic β-cells and amylase from pancreatic acinar cells are reduced. Furthermore, secretion of glucagon from pancreatic α-cells is improved [91]. Dipeptidyl-peptidase-IV (DPP-IV) (EC 3.4.14.5) from epithelial and endothelial cells inhibits the secretion of PYY and oxyntomodulin hormone from intestinal enteroendocrine cells and synthesis of intestinal incretin hormones (glucose-dependent insulinotropic polypeptide (GIP) from K cells of the upper intestine and GLP-1 from L cells of the lower intestine) responsible for postprandial insulin secretion [92]. CCK from the I cells of the gut decreases postprandial glucose levels and increases insulin levels in T2DM. Therefore, absence of CCK causes glucose intolerance and influences T2DM [93]. Deficiency of gastric leptin interrupts glucose homeostasis by modulating hepatic de novo gluconeogenesis and lipogenesis, and secretion of hepatic TG [94] as well as modulating the activity of insulin [95]. Adipose tissue releases glycerol, NEFAs, hormones (adiponectin, leptin and resistin) and proinflammatory cytokines (adipokines) that are involved in the development of insulin resistance [96]. Accumulation of excessive visceral fat may be the cause of dysfunctionality of adipose tissue and consequently increases the secretion of leptin and negatively affects adiponectin. Plasma leptin stimulates oxidative stress, inflammation and insulin resistance [97]. As a consequence of reduced insulin responsiveness in adipocytes, T2DM is associated with lipolysis through the downregulated expression of adipocyte lipid-droplet proteins, such as CIDE proteins [98] and perilipin [99]. Elevated plasma FFAs due to lipolysis may cause insulin resistance in obese individuals, which may lead to cellular uptake of FFA and enhance lipid oxidation. In muscle cells, FFA oxidation reduces insulin-mediated glucose disposal and in hepatic cells, it promotes gluconeogenesis and hepatic glucose output [11]. Postprandial hyperglycemia is associated with the activity of pancreatic α-amylase and α-glucosidase in the brush border of enterocytes of the jejunum in the small intestine. α-amylase is responsible for the initial hydrolysis of the α-D-(1-4) glycosidic bonds of polysaccharides and starch to oligosaccharides; and subsequently, oligosaccharides are hydrolyzed into monosaccharides by α-glucosidase. Therefore, their activity improves the rate of glucose absorption [100]. Insulin resistance encourages individuals to consume high-calorie foods, which is linked with gastric emptying and appetite. Food intake and energy expenditure are associated with lower activity of GLP-1, PYY, CCK and oxyntomodulin from the gut. Furthermore, ghrelin from the stomach, and leptin from the stomach and adipose tissues influence appetite activity [101].

**Figure 3:**
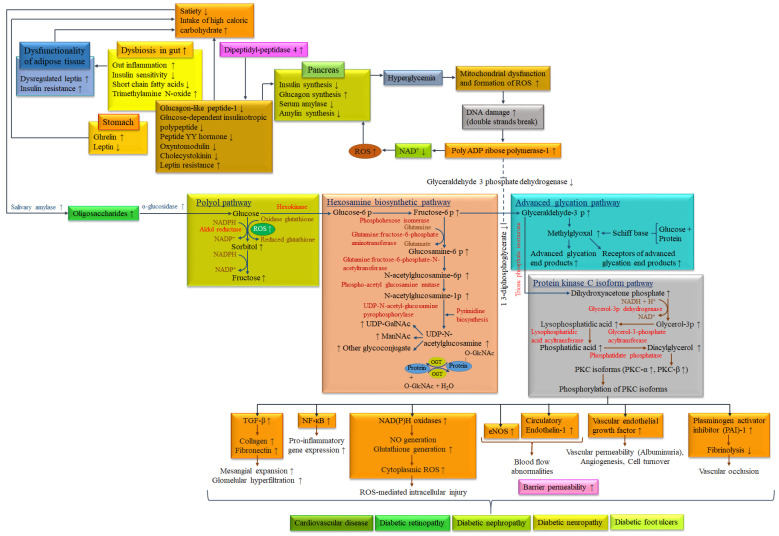
Metabolic pathways for diabetes and associated complications, induced by the consumption of high-calorie fast foods (self-developed, concept was adopted from Kang and Yang, 2020 [108], and Naveen and Baskaran, 2018 [109]).

There are a number of cellular stress-responsive metabolic pathways associated with hyperglycemia-induced superoxide synthesis and inflammation. Inflammation in the intestine, hepatocytes, adipocytes and pancreatic cells may influence the synthesis and abnormal regulation of these hormones and peptides, which may contribute to the development of diabetes. It has been proven that circulatory pro-inflammatory molecules, such as tumor necrosis factor (TNF)-α, interferon-gamma (IFN-γ), interleukin (IL)-1, IL-6, IL-8, IL-1β, transforming growth factor (TGF)-β, monocyte chemoattractant protein (MCP)-1, toll-like receptors (TLR)-2 and TLR-4 on the surface of monocytes, leptin, chemerin and plasminogen activator inhibitor (PAI)-1, retinol binding protein-4, C-reactive protein and monocyte chemotactic protein-1 are increased in diabetic and obese individuals. It may be supposed that these pro-inflammatory molecules are associated with systemic insulin resistance and T2DM complications [102]. In a hyperglycemic condition, a wide range of metabolic pathways, namely, the polyol pathway (formation of sorbitol from glucose and conversion to fructose), formation of AGEs pathway (formation of methylglyoxal from glucose and conversion to AGEs), protein kinase C (PKC) isoform pathway (glucose to diacylglycerol (DAG) and stimulation of PKC isoforms, especially PKC-β which subsequently phosphorylate) and hexosamine biosynthetic pathway (glucose to uridine diphosphate N-acetylglucosamine (UDP-GlcNAc) and subsequently N-acetylglucosamine (GlcNAc)) lead to both oxidative and endoplasmic reticulum stress, resulting in chronic inflammation and insulin resistance [103]. These metabolic pathways may cause a wide range of micro- and macro-vascular complications alongside diabetes, such as cardiovascular disease, stroke, peripheral vascular disease, retinopathy, nephropathy, neuropathy and foot ulcers [104]. Inflammation in the intestine is also associated with gut microbiota, which is related to the type of consumed foods and dietary patterns [105]. Dysbiosis reduces the formation of SCFAs and leads to the formation of trimethylamine N-oxide (TMAO). Bacterial translocation and their toxic metabolites lead to inflammation in the gut and reduce insulin sensitivity [106,107]. Putative relationships between different risk factors of diabetes and associated complications are quite complex. Biochemical activities of risk factors and their contribution to the metabolic pathways for diabetes and associated complications, induced by the consumption of high-calorie fast foods are presented in Figure 3.

**Figure 4:**
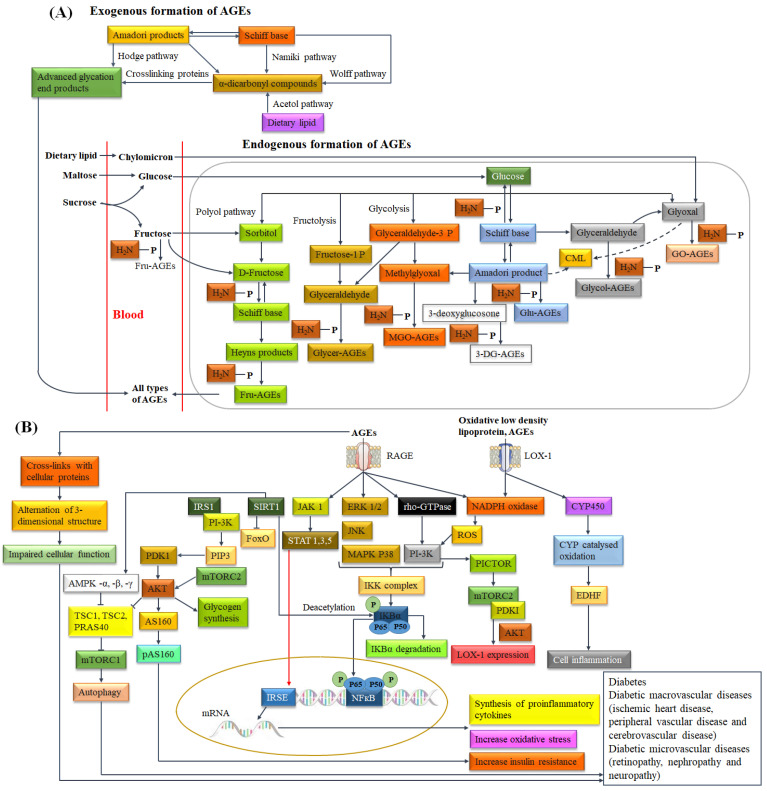
Biochemical pathways of (**A**) formation of exogenous and endogenous AGEs (self-developed, concepts were adopted from Inan-Eroglu et al., 2020 [124] and Takeuchi et al., 2020 [125]), and (**B**) AGE/RAGE-mediated signaling and metabolic pathways for DM (self-developed, concept was adopted from Salazar et al., 2021 [126] and Dong et al., 2023 [127]). Abbreviations: Fru-AGEs: Fructose-derived glycation end products, Glycer-AGEs: Glyceraldehyde-derived glycation end products, MGO-AGEs: Methylglyoxal-derived glycation end products, 3-DG-AGEs: 3-Deoxyglucosone-derived glycation end products, Glu-AGEs: Glucose-derived glycation end products, Glycol-AGEs: Glycol-derived glycation end products, GO-AGEs: Glyoxal-derived glycation end products.

Reactive carbonyl compounds (RCCs), such as AGEs are produced by non-enzymatic exogenous and endogenous systems and are recognized as key players in the progression of diabetes and diabetes-induced macrovascular and microvascular complications. The pathogenesis of carbonyl stress by AGEs is dependent on two pathways: (a) trapping and cross-linking of cellular or plasma proteins, including laminin, elastin and collagen with AGEs. It induces physiological dysfunction, such as alternation of elasticity and function of tissues, and (b) activating downstream cellular signaling pathways via RAGE receptors to trigger oxidative stress and inflammation [110]. AGEs may exist alone as an amino acid conjugate or with free low-molecular-weight (LMW, <5 kDa) peptides or bound with proteins. Absorption of pyrraline, a sugar derivative of Lys, is performed using peptide transporter 1 (PEPT1) across the intestinal epithelium [111]. The LMW peptide fractions of AGEs are more likely absorbed; while, non-absorbed AGEs are passed through the gastrointestinal tract [112]. AGEs can modulate cellular functions through binding with G-protein-coupled receptors (GPCRs), TLRs, scavenger receptors and pattern recognition receptors; however, the interactions between AGEs with cell surface receptor for advanced glycation end products (RAGE) is well-recognized for T2DM. Interaction between AGEs/RAGE influences an array of signaling events, such as activation of the renin–angiotensin system (RAS)-mediated extracellular signal-regulated kinase (ERK1/2), mitogen-activated protein kinase (MAPK), stress-activated protein kinase/c-Jun N-terminal kinase (SAPK/JNK), phosphoinositide 3-kinase/Protein kinase B (PI3-K/AKT), Janus kinase/signal transducers and activators of transcription (JAK/STAT), Ras homologous GTP-binding proteins (Rho GTPases) Rac-1, Cell division control protein 42 (Cdc42) pathways. Therefore, AGEs promote a wide range of cell-mediated pathophysiological responses, such as pancreatic β-cell toxicity, elevation of cytosolic ROS, activation of nuclear factor kappaB (NF-κB), increased formation of proinflammatory cytokines, stimulation of oxidative and endoplasmic reticulum stress. It may be realized that they are supposed to maintain mechanistic links between the risk factors of DM [110]. More specifically, the activation of JNK promotes the phosphorylation of insulin receptor substrate (IRS-1) at Ser residue, which leads to negative regulation of insulin signal transduction and induces insulin resistance. Activated NF-κB upregulates the expression of various inflammatory cytokines (TNFα, IL-1β, IL-6) and influences insulin resistance [113]. Interestingly, it has been reported that abnormal activation of the ERK1/2 signaling pathway is associated with several diabetogenic factors and adipogenesis [114]. Interaction between AGE and RAGE modulates intracellular cascade of biochemical reactions which inhibits insulin-induced GLUT-4 translocation and induces insulin resistance [115]. RAGE binds with toxic intermediates from amyloid polypeptide (IAPP) and transduces intracellular signals that lead to NADPH oxidase-mediated cellular stress and inflammation. It plays a significant role in pancreatic amyloidosis-induced β-cell proteotoxicity and β-cell apoptosis [116]. The accumulation of AGEs in the endoplasmic reticulum interferes with protein folding. Furthermore, AGEs influence mitochondrial proteins in the respiratory chain and may cause mitochondrial dysfunction (reduces the synthesis of adenosine triphosphate (ATP)) which may cause oxidative stress [117]. When AGEs in the body are relatively high, AGE-R1 typically degrades AGEs in cells and influences receptor-mediated endocytosis. AGE-RI inhibits RAGE-mediated activation of the proinflammatory gene NF-κB through upregulation of SIRT1 and subsequently inhibits oxidative stress [118]. AGE-modified low-density lipoprotein (LDL) contributes to reduced LDL clearance and oxidized LDL via activation of *LOX-1*. Oxidized LDLs exhibit a wide variety of biological and atherogenic properties involving the activation of inflammatory and mitogenic pathways. The biological activity of oxidized LDLs depends on the presence of lipid peroxidation products, such as methylglyoxal. Modified LDL alters the affinity with apoB/E receptor and deviates the metabolism towards scavenger receptor-bearing cells, such as macrophages and smooth muscle cells. It was reported that the formation of atherogenic foam cells from smooth muscle cells with angiotensin II is progressive in T2DM [119]. Activation of *LOX-1* along with oxidized LDL or AGEs can produce ROS through the activation of NADPH oxidase, which subsequently influences the ERK1/2-MAPK and PI3K pathway [120]. Interaction between AGE and RAGE activates PI3K/PDKI along with downstream phosphorylation of mTOR, which triggers the mTOR pathway [121]. Furthermore, *LOX-1* influences cytochrome P450 (CYP450) which modulates the activity of endothelium-derived hyperpolarizing factor (EDHF) and subsequently may cause inflammation [122]. Unabsorbed AGEs in the colon can affect the homeostasis of intestinal microbiota, specifically the loss of butyrate-producing bacteria. The damage to the colonic epithelial barrier and chronic low-grade inflammation due to the loss of butyrate-producing bacteria was correlated with insulin resistance and the pathogenesis of DM [123]. Biochemical pathways and metabolites associated with the consumption of fast foods are presented in Figure 4.

### 3.2. Dyslipidemia

The metabolic disorder dyslipidemia is the imbalanced formation of elevated levels of TG or LDL-C or lower formation of HDL-C. It may be caused by (a) mutations of a single gene or multiple genes, (b) intake of excessive dietary saturated fats and soluble carbohydrates (glucose, sucrose, fructose) with high caloric index, (c) over consumption of alcohol and cigarette smoking, (d) diseases, including diabetes mellitus, chronic kidney disease, hypothyroidism, HIV infection, nephrotic syndrome, primary biliary cirrhosis and other cholestatic liver diseases, and (e) intake of medications and anabolic steroids [128]. It was shown that the prevalence of dyslipidemia in pediatrics may be associated with high BMI and promotes other cardiometabolic risk factors, such as insulin resistance, high blood pressure and endothelial dysfunction. Therefore, dyslipidemia in childhood is associated with atherosclerosis in adulthood [129].

Dyslipidemia is traditionally classified by patterns of elevation in lipids and lipoproteins in plasma, such as (a) pure or isolated hypercholesterolemia, which is caused by an increase in cholesterol only, (b) pure or isolated hypertriglyceridemia, which is caused by an increase in TGs only, and (c) mixed or combined hyperlipidemias, which is caused by an increase in both cholesterol and TG [130]. Systematically dyslipidemia may be categorized by the Fredrickson phenotype, such as (a) phenotype I where the elevated lipoprotein is chylomicron and the elevated lipid is TG, (b) phenotype IIa* where the elevated lipoprotein is LDL-C and the elevated lipid is cholesterol, (c) phenotype IIb* where the elevated lipoproteins are LDL-C and VLDL, and the elevated lipids are TG and cholesterol, (d) phenotype III where the elevated lipoproteins are VLDL and chylomicron remnants, and the elevated lipids are TG and cholesterol, (e) phenotype IV where the elevated lipoprotein is VLDL and the elevated lipid is TG, and (f) phenotype V where the elevated lipoproteins are chylomicrons and VLDL, and the elevated lipids are TG and cholesterol [131]. Particularly high levels of plasma LDL-C are considered as a major risk factor for CVDs; whereas hypertriglyceridemia associated with other diseases may cause acute pancreatitis and NAFLD. Dyslipidemia could be determined genetically (primary or familial dyslipidemia) or secondary to other conditions including DM, an unhealthy lifestyle, and so on. Hypercholesterolemia with elevated plasma LDL-C is the most common form of dyslipidemia and it increases the risk of CVDs. The global burden of dyslipidemia has significantly increased over the last 30 years. Hypercholesterolemia was considered the 15th leading risk factor for death in 1990 and 8th in 2019 [132].

Primary lipogenic tissues are hepatocytes; however, the contributions of white adipose tissue (WAT) and brown adipose tissue (BAT) are well recognized for TG homeostasis and energy storage [133]. Hepatic TG may be derived from dietary intake of long, medium and free fatty acids, esterification of plasma FFA and hepatic DNL. Fasting and postprandial TG concentrations are significantly increased by hepatic DNL [134]. In hepatocytes and adipocytes, a wide range of transcription factors (liver X receptor (LXR), sterol regulatory element-binding protein-1c (SREBP-1c) and carbohydrate response element binding protein (ChREBP)) and lipogenic enzymes (acetyl-CoA carboxylase, fatty acid synthase, fatty acid elongase 6 and stearoyl-CoA desaturase 1) play a significant role in DNL. The biochemical pathway of hepatic lipogenesis may be characterized by three sequential steps, such as fatty acid synthesis (formation of palmitate), fatty acid elongation (formation of stearate from palmitate)/desaturation (formation of oleate from stearate) and esterification (formation of TG) [135]. Therefore, saturated fatty acids (SFA) through dietary intake are associated with DNL and obesity. The consumption of dietary SFA can promote insulin and leptin resistance, and inflammation via endoplasmic reticulum stress, cellular apoptosis and activation of proinflammatory pathways in the hypothalamus, hepatocytes, adipocytes, skeletal muscle, pancreas and gastrointestinal tract [136]. Interestingly, it was reported that dietary fructose increases insulin resistance and levels of enzymes involved in DNL even more than a high-fat diet [137]. Fructose is absorbed via the portal vein because it does not require insulin for its metabolism, a single phosphorylation step by fructokinase to form fructose-1-phosphate prior to conversion to dihydroxyacetone phosphate (DHAP) and glyceraldehyde (GA) and non-influenced activity of phosphofructokinase by ATP and citrate. Therefore, fructose is delivered to hepatocytes in much higher concentrations, unlike glucose, and induces DNL. Interestingly, it has been reported that prolonged consumption of fructose may lead to hyperinsulinemia and insulin resistance. Furthermore, fructose metabolism is associated with the depletion of ATP, suppression of fatty acid oxidation in mitochondria, formation of uric acid and endoplasmic reticulum stress, which promotes inflammation and the formation of ROSs [138].

**Figure 5:**
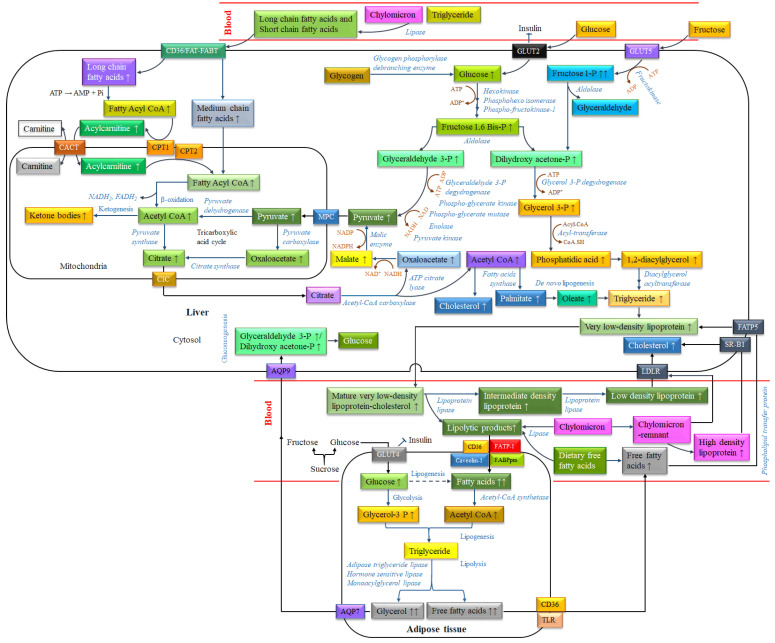
Metabolic pathways for dyslipidemia caused by soluble carbohydrates (glucose and fructose) and dietary fats in high-calorie fast foods (self-developed, concept was adopted from Fukushima et al., 2015 [154], and Mato et al., 2019 [155]).

DNL is physiologically regulated, where a coordinated series of enzymatic metabolic reactions are involved. For example, during fasting, DNL is downregulated due to high levels of blood glucagon and cellular cAMP. On the other hand, blood glucose and insulin levels are improved after consumption of a carbohydrate diet. Those stimulate DNL through increasing substrate availability, activity of lipogenic enzymes and expressions of lipogenic genes [139]. Glucose and fructose are directly absorbed in the luminal membrane of enterocytes in the intestine via an energy-independent mechanism. Intestinal fructose is transported by glucose transporter 5 (GLUT5) on the lumenal side and glucose transporter 2 (GLUT2) on the basolateral side [140]. Furthermore, enterocytes of the small intestine play a significant role in the absorption of chylomicron from dietary fatty acids which are subsequently transported by carnitine palmitoyltransferase (CPT) and carnitine-acylcarnitine translocase (CACT) in the mitochondrial membrane [141]. The biochemical process of DNL in hepatocytes begins with the formation of DHAP and glyceraldehyde 3-phosphate (GA3P) from soluble dietary carbohydrates. The formation of triose-phosphates (GA, DHAP, GA3P) may concomitantly increase the formation of methylglyoxal (MG) as well as a precursor for AGEs, which have been recognized in the pathogenesis of T2DM [142]. Triose-phosphates are further metabolized to pyruvate and transported to mitochondria by MPC, a mitochondrial pyruvate transporter for energy production. In hepatic mitochondria, pyruvate is oxidized to acetyl-CoA and subsequently used in the tricarboxylic acid (TCA) cycle. When energy accumulation is abundant, citrate is transported from the mitochondria to cytoplasm of hepatocytes by the mitochondrial citrate/isocitrate carrier (CIC). Citrate is converted to acetyl-CoA, which is the first step of endogenous lipogenesis. Palmitate (C16:0) is the primary endogenously synthesized fatty acid; however, it may be elongated to stearate (C18:0) and subsequently to oleate (C18:1). The final step of lipogenesis is the conversion of diacylglycerols into TG, catalyzed by diacylglycerol acyltransferase (DGAT). In hepatocytes, TG is stored as stored energy depots or assembled into VLDL and subsequently transported to the blood. Hepatic DNL induces fasting and postprandial TG concentrations [134]. VLDL in the bloodstream is converted to LDL and lipolytic products via the complex catalytic activity of lipoprotein lipase. LDL enters hepatocytes via LDL receptor (LDLR) and LDLR-related protein-1 (LRP1), and acts as a precursor of TG [143]. Circulating glucose/fructose, fatty acids from dietary chylomicrons, dietary FFAs and VLDL derivatives from hepatocytes are initiators of DNL in adipocytes. Fatty acid derivatives enter adipocytes through fatty acid transporters, such as fatty acid transport protein-1 (FATP1), cluster of differentiation 36 (CD36), Caveolin-1 and FABPpm [144]. DNL in adipose tissue is decreased with increasing insulin resistance [145]. Therefore, in diabetic conditions, adipose-specific GLUT4 is downregulated and TG is formed by lipolytic products from blood in adipocytes by esterification [139]. It has been proven by several clinical investigations that DNL is significantly higher in hepatocytes than adipocytes due to carbohydrate-rich diets [146]. Hydrolysis of TG (lipolysis) by adipose triglyceride lipase (ATGL), hormone-sensitive lipase (HSL) and monoacylglycerol lipase (MGL) in adipose tissues liberates FFA and glycerol, which can be taken up by hepatocytes and converted to VLDL in both fed and fasted states. In the first step, ATGL hydrolyzes TG into diacylglycerol (DAG) and the first molecule of fatty acid. Subsequently, HSL hydrolyzes DAG into monoacylglycerol (MAG) and the second molecule of fatty acid. Finally, MGL hydrolyzes MAG into glycerol and the third molecule of fatty acid. These liberated fatty acids can be oxidized in BAT or muscle and glycerol may be used as a precursor in gluconeogenesis in hepatocytes [147]. Increased FFAs from lipolysis in adipose tissue reduces β-oxidation within the liver and upregulates hepatic DNL [148]. It has been proven by several clinical investigations that a higher proportion of hepatic TG is derived from plasma FFA, produced by lipolysis in adipose tissues than hepatic DNL in subjects with normal liver fat [149,150]. Under pathological conditions, lipolysis in WAT leads to lipotoxicity and insulin resistance [151]. Furthermore, lower lipogenesis in WAT decreases the synthesis of insulin-sensitizing fatty acids and consequently insulin resistance [152]. Due to the consumption of fast foods, the amount of caloric intake exceeds the calorie demand. Adipose tissues accumulate TG and grow both in size and number, resulting in obesity. The accumulation of diacylglycerols in the pathway of DNL induces insulin resistance by activating PKC, the transcription factor of NF-κB and c-Jun N-terminal kinase 1, which is linked with diabetes. Furthermore, hyperinsulinemia promotes lipolysis and the release of FFA from adipocytes, which is the cause of imbalance of adipocytokines (decreased adiponectin and/or increased proinflammatory cytokines) and lipotoxicity, and consequently hepatic steatosis [153]. Interestingly, some recent investigations represent that adipocyte-specific fatty acids improve systemic insulin sensitivity and decrease inflammation [145]. Metabolic pathways related to dyslipidemia due to the consumption of soluble carbohydrates (glucose and fructose) and dietary fats in high-calorie fast foods are presented in Figure 5.

Dyslipidemia may lead to symptomatic end-organ diseases, including vascular disease (coronary artery disease (CAD), stroke and peripheral arterial disease), acute pancreatitis and hepatosplenomegaly. Very high TG levels may cause dyspnea, paresthesias and confusion [156]. Severe hypertriglyceridemia (>22.6 mmol/L) may cause lipemia retinalis. Severe dyslipidemia may include localized lipid deposits (xanthoma). High levels of LDL-C may cause arcus cornea and xanthelasma palpebrarum. Xanthelasma palpebrarum can also occur in patients with primary biliary cirrhosis. Furthermore, high LDL-C levels may cause tendinous xanthomas at the achilles elbow, and knee tendons and over metacarpophalangeal joints [157].

## 4. Management of Obesity

Obesity has been acknowledged as a major challenge in the healthcare system and is considered an economic burden around the world. Reduction in the prevalence and risk of obesity involves a combination of lifestyle changes and pharmacological interventions. Reduction in body weight and maintaining the ideal body weight are essential to prevent a wide range of metabolic diseases. The reduction in body weight signifies a loss of 7–10% in baseline body weight within 6–12 months. It is associated with a reduction in caloric intake by 500–1000 calories·day^−1^ [1,158]. Management of obesity includes cooperation between patients and healthcare professionals with different specializations, including dietetics, endocrinologists, medicine specialists, psychologists and physiotherapists in order to achieve the best possible treatment outcome [159]. Three medications, such as sibutramine, orlistat and rimonabant are generally used for weight loss. Sibutramine, a monoamine-reuptake inhibitor, results in average weight losses of 4–5 kg; however, this medication is not suitable for patients with coronary heart disease. Orlistat, a gastrointestinal lipase inhibitor, reduces weight by average 3 kg and decreases development to diabetes in high-risk patients; however, adverse gastrointestinal disorders were noted. Rimonabant, an endocannabinoid receptor antagonist, reduces weight by an average of 4–5 kg and waist circumference. It improves the concentrations of plasma TG and HDL-C; however, an incidence of mood-related disorders can be noted [160]. Unfortunately, the unavailability of a single medication for pharmacotherapy of metabolic diseases including obesity and prolonged use of multiple medications are considered as challenging issues due to the high risk of side effects [161]. Bariatric surgery has been considered an effective treatment for metabolic diseases including obesity due to a significant decrease in calorie intake. Dramatic improvement occurs in the case of glucose intolerance/insulin resistance one year after the surgery in patients [162]; however, some bariatric surgery risks include acid reflux, anesthesia-related risks, dizziness, chronic nausea and vomiting, dilation of the esophagus, intestinal infection, ulcers, bowel obstruction, hernias and low blood sugar [163]. Low-calorie diets and physical activity are generally recommended by dietitians and medical practitioners as a first-line treatment for managing obesity and are approved by most dietary guidelines and scientific societies. For a long time, physical exercise [164,165], intake of healthy diets [166,167], natural products, and herbal medicines were considered for individuals with such risk factors [168,169]. Current guidelines for the management of T2DM recommend progressive resistance training (PRT) to improve muscle mass and glycemic control [170]; however, nutritional/diet management is also considered a central element for the treatment of T2DM and dyslipidemia. Presently, multiple studies have confirmed the beneficial effects of the Mediterranean diet (fruits and vegetables, whole grains, healthy fats, etc.) on obese individuals [171,172].

### 4.1. Nutraceuticals and MS

Understanding human physiology behind weight and energy gain through food intake is extremely relevant to controlling obesity incidence and other metabolic diseases. Nutritional challenges as well as caloric restriction for the management of metabolic diseases have been reported several times [173]. Therefore, there is growing interest in nutraceuticals/functional foods, management of diet habits, and lifestyle for reducing the risk factors of metabolic diseases. It may be realized that the selection of unique functional foods provides wide ranges of biological activities, which can modulate different metabolic pathways associated with the risk factors of MS [174]. A variety of plant-based nutraceuticals and their contributions to metabolic diseases have been reported. Some examples of them are mentioned herein: curcumin from turmeric (*Curcuma longa*), allicin from garlic (*Allium sativum*), quercetin from onion (*Allium cepa* L.), gingerols, shogaols, and parasols from ginger (*Zingiber officinale*), cuminaldehyde from cumin (*Cuminum cyaminum*), terpenine and cineol from cardamom (*Elettaria cardamomum*), polyphenols from cinnamon (*Cinnamomum verum*), neem oil from neem seed (*Azadirachta indica*), berberine from *Rhizoma coptidis*, bergamot essential oil from bergamot orange (*Citrus bergamia*), resveratrol and 3,5,4′-trihydroxy-*trans*-stilbene from grape seed (*Vitus vinifera* L.), saponins and galactomannan from fenugreek (*Trigonella foenum*), polyunsaturated fatty acids from animal- and plant-based oils, sulforaphrane from broccoli (*Brassica oleracea*), and SCFAs from symbiotic activity of prebiotics and probiotics [158,175].

Some recent investigations indicate that diets rich in protein and vegetables with some dietary restriction, such as consumption of refined carbohydrates can reduce insulin resistance irrespective of the change in body weight [162]. The unique nutritional value of dairy milk has catapulted it to the forefront of the functional food sector. For a long time, dairy milk has been well accepted in diet charts for all ages around the globe. The presence of wide ranges of biomolecules, such as proteins, carbohydrates, fats, minerals, and vitamins in milk offers unique nutritional value to consumers of all ages [176]. Several epidemiological and cohort studies confirmed that the consumption of dairy products decreased the prevalence of MS, while experimental studies pointed to the roles of dairy protein [177] and peptides [178] as dietary components that may modulate the activities of risk factors of MS. Potential beneficial outcomes from milk proteins include a wide range of aspects. Protein generally increases satiety to a greater extent than other primary macromolecules (carbohydrates and fats) and may facilitate a reduction in energy consumption under all dietary conditions. It may be supposed that the metabolic and appetite-suppressing effects of proteins are dependent on the quality of the protein, which is determined by the amino acid composition (non-essential or essential, glucogenic or ketogenic). Diets with higher amounts of protein are associated with increased thermogenesis, which influences the synthesis of satiety hormones, decreasing dietary calorie consumption and improving energy expenditure. Consequently, protein may provide a stimulatory effect on muscle protein anabolism and maintain BMI. Furthermore, peptides with unique functional values are produced by in vivo gastrointestinal digestion of dietary proteins. Their unique characteristics modulate physiological functions in an inclusive way and reduce the risks of a wide range of diseases [179].

### 4.2. Proteins and Peptides from Milk Proteins

According to the FAO dairy review, ~930 million tons of milk was produced around the globe in 2022, and it is expected to increase by 1.7% annually for almost a decade from now. Cows are the largest global milk source (81%), followed by buffalo (15%). On a global level, milk consumption stands at 112 kg·person^−1^·year^−1^, and milk and dairy products contribute about 18–20% of protein consumption in adults [180]. Therefore, peptides from cow and buffalo milk proteins, represented by dairy milk proteins for the management of obesity, are considered in the present review article.

Cow milk and buffalo milk contain ~3.6% and ~4.3% protein by weight, respectively [181], and have been considered excellent sources of all types of amino acids, including essential amino acids (EAAs). The protein digestibility-corrected amino acid score (PDCAAS) and digestible indispensable amino acid score (DIAAS) of milk proteins are higher than those of most other protein sources [182]. Dairy proteins are classified as (a) casein protein (80% (weight basis): αs_1_-casein, αs_2_-casein, β-casein, κ-casein) and (b) whey protein (20% (weight basis): β-lactoglobulin, α-lactalbumin, immunoglobulins (IgG, IgA, IgM), bovine serum albumin, lactoferrin, lactoperoxidase). The minor proteins in milk are glycoproteins and lipoproteins, which account for less than 2% of total milk proteins. They are produced from distinct genes. Buffalo milk is just like cow milk and contains two major protein fractions, such as casein protein and whey protein. Buffalo milk has a higher protein content than bovine milk. Therefore, it contains higher concentrations of caseins and whey proteins than bovine milk. The characteristics and quantity of milk proteins may change depending on the breed, feeding regime, seasons, and rearing system of animals [181]. Different distributions of caseins (α_s1_-casein, α_s2_-casein, β-casein, and κ-casein) and major whey proteins (β-lactoglobulin and α-lactalbumin) in cow milk and buffalo milk are reported in Table 2.

Numerous attempts have been made to characterize milk proteins [184] and their biological activities [185]. Casein contains a higher proportion of different types of EAAs, such as His, Met, Phe, and Val, than whey proteins. Furthermore, casein contains a higher proportion of several non-EAAs, including Arg, Glu, Pro, Ser, and Tyr. On the other hand, whey protein contains a higher proportion of the branched-chain amino acids (BCAAs), such as Leu, Ile, and Val, compared to casein. However, casein and whey proteins contain all EAAs, but their digestion and absorption characteristics are different. Whey proteins are highly soluble in the food matrix and under in vivo conditions. Therefore, after gastrointestinal digestion of whey proteins, their absorption rate is much faster than that of digested casein, which leads to a dramatic short-lived rise in plasma AAs. On the other hand, casein clots in the acidic conditions of the stomach, which leads to delayed gastric emptying and a steady and prolonged release of AAs into the bloodstream [186,187,188]. Therefore, whey protein is considered to be nutritionally superior to casein in terms of biological value, protein efficiency ratio, and net protein utilization. For example, the biological values for whey proteins and casein are 104 and 77, respectively; the protein efficiency ratio values for whey proteins and casein are 3.2 and 2.5, respectively; and net protein utilization for whey proteins and casein are 92 and 76, respectively [186,189]. Furthermore, the composition of amino acids and protein/food structure may play an important role in protein-stimulated physiological and metabolic effects. For example, casein could attenuate plasma postprandial glucose with nominal stimulation of insulin secretion, whereas the improvement of insulin response after whey causes a greater reduction in plasma postprandial glucose [190,191].

Besides the advantageous outcomes from dairy milk proteins, epitope mapping studies presented the sequential or linear and conformational epitopes within milk proteins. Due to the presence of immunoglobulin E (IgE)- and immunoglobulin G (IgG)-binding epitopes in the protein structure, dairy milk proteins are listed among the “big 8” allergens. Therefore, dairy milk protein allergy can be attributed to IgE-mediated and non-IgE-mediated mechanisms [192]. Dairy milk protein-allergic subjects could be segregated into different phenotypes according to reactivity [193]; however, no specific structure and function of dairy milk proteins account for a major part of the allergenic activity of dairy proteins. Therefore, it may be realized that the heterogeneity of the human IgE response could be attributed to the allergenic potential of any dairy milk protein or protein fragment [194]. Clinical polysensitization (cross-sensitization or cross-reactivity and co-sensitization) to several dairy milk proteins is most often noted. IgEs and IgGs from mice allergic to cow milk are capable of cross-reacting with buffalo milk proteins due to homologies in the composition of amino acids [195]. Some major characteristics of different types of dairy milk proteins are mentioned in Table 3.

**Figure 6:**
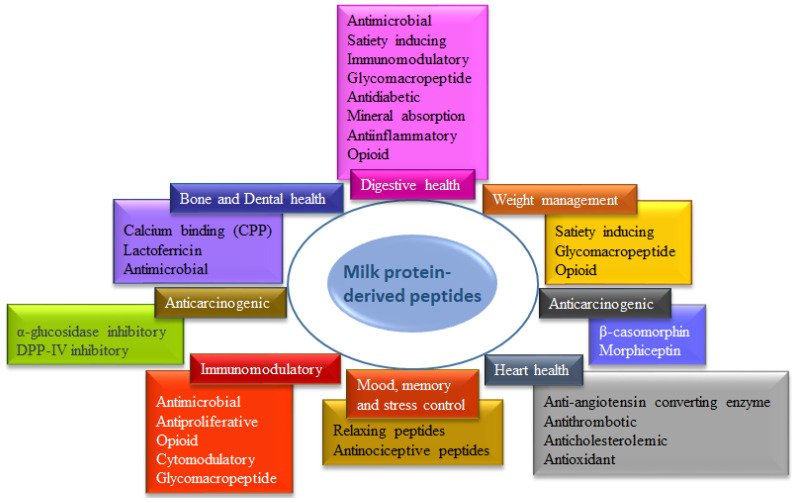
Physiological functions of milk protein-derived peptides (self-developed, concept was adopted from Park and Nam, 2015 [202]).

Apart from being an excellent source of EAAs for human nutrition, milk proteins can exhibit a wide range of biological activities by producing peptides due to gastrointestinal digestion. Peptides from dairy milk proteins are recognized as antioxidant, anti-angiotensin, antimicrobial, antidiabetic, anticarcinogenic, anti-inflammatory, antihypertensive, antihypercholesterolemic, immunomodulatory, and many more [185,201]. These advantageous outcomes from dairy milk proteins have encouraged the production of a wide range of dried protein powders, such as milk protein concentrate (MPC), milk protein isolate (MPI), whey protein concentrate (WPC), and whey protein isolate (WPI), as well as regular dairy foods, such as yogurt, kefir, and ice cream. Different physiological functions of dairy milk protein-derived peptides are shown in Figure 6.

In the last two decades, the attention of the dairy industry has shifted. Presently, the applications of dairy milk proteins are not limited to the development and fortification of regular dairy foods. The significant income from individual dairy proteins and peptides has been taken into consideration along with the marketing of regular dairy foods [203]. It is well recognized that peptides from milk proteins modulate the synthesis and activities of different risk factors of MS-associated diseases [204,205]. Some milk protein-derived specific peptides offer two or more distinct biological activities (multifunctional activities) [206]. Peptides are protein fragments produced by the action of proteolytic enzymes and generally contain 2–20 amino acid residues and generally have a molecular weight < 6 kDa [207]. Hydrolysis of milk proteins can be performed using suitable acids or alkalis or food-grade proteolytic enzymes. Enzymatic hydrolysis of dairy milk proteins is preferred because acid and alkali hydrolysis of milk proteins is difficult to control. Acid hydrolysis oxidizes Cys and Met and partially destroys Ser and Thr and converts Gln and Asn to Glu and Asp acids, respectively. Furthermore, alkali hydrolysis of milk proteins causes racemization in amino acids. Enzymatic hydrolysis of dairy milk proteins has been recognized as “safe” by the European Food Safety Authority (EFSA) and the FDA [208]. Peptides from both plant- and animal-based proteins can be produced by a wide range of proteases under in vitro conditions, and by fermentation and ripening by microbial proteolytic systems. Furthermore, they can be produced in the gastrointestinal tract (in vivo conditions) by digestive enzymes, such as pepsin, chymotrypsin, and trypsin. Proteolysis of milk proteins through enzymatic routes not only produces peptides with unique functional activities but also reduces the allergenic activity of proteins and oligopeptides [209]. Dairy milk proteins α-casein and β-casein have the potential to liberate more than 20,000 functional peptides each [210]. The first identified food protein-derived peptide was from casein, which had the potential for vitamin D-independent bone calcification in rachitic infants [211]. Intestinal absorption of peptides is supposed to follow any of the following mechanisms: carrier-mediated transporter system, passive diffusion and transcytosis, paracellular pathways through tight junctions, and endocytosis [212]. One of the shortcomings of producing peptides from milk proteins by enzymatic hydrolysis is the generation of peptides with a bitter taste [213]. Some well-recognized peptides produced by in vitro enzymatic hydrolysis of dairy milk proteins and their functional activities against metabolic risk factors are presented in Table 4.

Unfortunately, adequate quantities of peptides are not produced through the enzymatic hydrolysis of food proteins, including dairy milk proteins. Therefore, this technique is not always considered economical. Chemical and recombinant DNA technologies came to the forefront to solve the mentioned problem. Chemical synthesis is preferred for producing small and medium-chain-length peptides, usually composed of 5–80 amino acids in sequence, and recombinant DNA technology is preferred for producing larger peptides and proteins. These techniques are usually employed when the peptide sequence is known [239]. Chemical modifications of peptides usually improve membrane permeability, affinity with receptors, and stability during delivery. Furthermore, it has been proven that the bioavailability of peptides is improved by chemical modification. Purification of such synthesized peptides from other products in the reaction mixture is a considerably challenging issue. Gene expression in microorganisms using modern recombinant DNA technology and cloning are advantageous for synthesizing peptides; however, these technologies require intensive research [240]. Bovine β-casomorphin 7 (BCM-7), which has the potential to prevent cardiovascular diseases, type I diabetes, and neurological disorders, was synthesized through this advanced molecular biology technique [241].

Currently, attempts are being made to assess the biological significance of milk protein-derived peptides and their characterization through advanced technologies and concepts, known as peptidomics [242]. In the last few decades, the BIOPEP database [243] has been upgraded. The outstanding development of peptidomics [242] opens a new horizon in science, known as nutrigenetics and nutrigenomics [244]. Recently, they are being considered in dairy foods to understand the biofunctional activities in a comprehensive way [245]. Presently, in silico approaches, including quantitative structure–activity relationship (QSAR), are used to predict the physicochemical information of these peptides and their responsive biological activities [246].

Peptides from food proteins, including dairy milk proteins, have gained attention in both the nutraceutical and pharmacological sectors due to their unique therapeutic potential and biocompatibility without side effects. In some cases, they are used for the fortification of conventional foods to provide unique beneficial outcomes to consumers [247,248]. Dietary products enriched with milk protein-derived peptides are commercially available with therapeutic information [249]. Some of them are used for combating metabolic diseases. Commercial peptides from dairy milk proteins, along with their sequence and appealing health or functional benefits, are mentioned in Table 5.

#### 4.2.1. Anti-Diabetic Activity

Both casein and whey protein-derived peptides or amino acids may affect insulin secretion from pancreatic β-cells and the release of incretin hormones, i.e., GIP and GLP-1, from the gut. It is anticipated that insulin sensitivity is related to the characteristics of the amino acid pool (qualitative and quantitative) from dairy proteins in plasma [250]. Whey protein is more effective than micellar casein for the rapid secretion of insulin [187]. It was found that the absorption rate of casein in its native micellar form is lower [251]; however, hydrolysis of casein improves the absorption of amino acids and the secretion of insulin [252]. The insulinotropic activity of WPH might be related to intestinal amino acid absorption and the increased concentration of FAAs (Leu, Ile, Phe, Arg, Tyr, Thr, Val, Ala, and Lys), BCAA-containing dipeptides (IL, LL, and VL), and possibly cyclic dipeptides in plasma [253,254]. Furthermore, whey proteins influence the synthesis of GLP-1 more than casein [255]. Insulin does not have a remarkable contribution in insulin-sensitive tissues exclusively; it has wide-ranging direct and indirect effects on metabolism, including stimulation of glucose uptake, glycogen synthesis, gluconeogenesis, lipid uptake, TG synthesis, lipolysis, protein synthesis, and inhibition of protein breakdown [256]. In addition to stimulating insulin release from pancreatic β-cells, dairy proteins and their hydrolysates may alter tissue glucose uptake in skeletal and muscle cells and suppress the high postprandial blood glucose level [257,258]. Interestingly, it was proven that longer-term consumption of casein and whey protein supplementation decreases fasting blood insulin levels and diminishes the physiological effects of DM [259]. The contribution of milk protein-derived peptides with anti-DPP-IV activity and anti-α-glucosidase and anti-α-amylase activity in hyperglycemic conditions was anticipated by several investigators [260,261]. Both hydrophobic amino acids (Ala, Gly, Ile, Leu, Phe, Pro, Met, Trp, and Val) and hydrophilic amino acids (Thr, His, Gln, Ser, Lys, and Arg) are found within DPP-IV inhibitory peptides. The presence of hydrophobic amino acids in potent DPP-IV inhibitory peptides may enhance the interaction with the active site of DPP-IV (S1 subsite of DPP-IV) [262,263]. Peptides from dairy milk proteins with one or two Pro residues at the N-terminus are highly effective DPP-IV inhibitors; however, tryptophan-containing milk protein-derived dipeptides against DPP-IV were reported [264]. Casein, particularly β-casein, contains a high amount of Pro residues. Therefore, peptides from β-casein are potential DPP-IV inhibitors compared to β-lactoglobulin-derived peptides [265]. It is anticipated that two other enzymes, α-amylase and α-glucosidase, play significant roles in normal glucose homeostasis. α-Amylase competitively interacts with peptide sequences having hydrophobic amino acids, such as Leu, Met, and Pro, at the terminal; while α-glucosidase is inhibited by the hydrophobic amino acids Met, Pro, Phe, and Leu in peptide sequences [228]. It has been proven that antioxidant peptides offer antidiabetic activity and can protect cells from reactive oxygen-induced stress by modulating multiple pathways and biochemical reactions, including inhibition of the synthesis of malondialdehyde (MDA) and protein carbonyls [266], reduction in the activity of lactate dehydrogenase (LDH) [267,268], increase in the activity of oxidative enzymes (catalase, glutathione peroxidase, and superoxide dismutase) [269], and expression of genes in the Keap1-nuclear factor erythroid 2–related factor 2 (Nrf2) signaling pathway [267,270]. Peptides with effective antioxidant capacity are hydrophobic in character and comprise unique amino acids, such as Trp, Tyr, His, and Pro. In addition, the presence of two hydrophobic amino acids, such as Leu and Val, in peptides contributes to antioxidant and lipid peroxidation capacity [266]. Two well-known milk protein-derived peptides, IPP and VPP, demonstrated effective enhancement of insulin signals and anti-inflammation via the NF-κB pathway under TNF stimulation and prevention of insulin resistance. These two peptides offer an insulin-sensitizing effect, which is independent of insulin receptors in adipocytes. Furthermore, they are recognized as potential DPP-IV inhibitors [234,271].

In addition, peptides from casein and whey protein show a satiating effect [272], which is related to the gastrointestinal system and the appetite center of the brain [273]. Casein reduces gastric emptying and improves a slow postprandial increase in amino acids in plasma. Contradictorily, whey proteins improve a fast, high, and transient increase in amino acids in plasma. Some investigations have suggested that whey proteins are more satiating than casein proteins [255,274], and their thermogenesis and influence on body weight differ to some extent [275,276]. Casein hydrolysates activate peripheral opioid and CCK receptors and block the antagonist receptors associated with food consumption [277,278]. Whey protein-derived peptides increase the concentration of postprandial plasma amino acids, CCK, GLP-1, and GIP. The satiating effect of whey protein is mainly due to a high concentration of BCAAs, particularly L-Leu [279]. Therefore, milk protein-derived peptides modulate the release of satiety hormones (CCK, GLP-1, GIP, PYY, an ghrelin), increase diet-induced thermogenesis, and activate opioid receptors [190]. Milk protein-derived small peptides and amino acids act via GPCRs, increasing intracellular Ca^2+^ and/or the concentration of cyclic adenosine monophosphate (cAMP), or via peptide/amino acid transporters, which depolarize the enteroendocrine cell membrane, activating Ca^2+^ influx and stimulating satiety hormones [280,281].

#### 4.2.2. Anti-Dyslipidemic Activity

Dairy milk proteins modulate intrahepatic lipids and circulating TGs through a wide range of biochemical mechanisms, including gastric emptying, insulin secretion, lipogenesis (downregulation), and gluconeogenesis and glycogenesis (upregulation) in hepatocytes. They suppress postprandial lipemia due to insulinotropic effect, modulate metabolic pathways dedicated to lipid metabolism, and offer lipid peroxidation activity [282].

The concentration of postprandial TG is influenced by many factors, such as insulin concentration, secretions of intestinal chylomicrons and hepatic VLDL, transformation of TG-rich lipoproteins to TG-depleted lipoproteins, and tissue uptake of TG-depleted lipoproteins [283]. Insulin is a well-known activator of lipoprotein lipase, but high levels of postprandial insulin inhibit hormone-sensitive lipase [284]. Therefore, consumption of dairy milk proteins may result in lower formation of chylomicrons, and subsequently, FFA from adipose tissue and lipotoxicity in hepatocytes. Due to the insulinotropic effect of whey proteins, they may enhance the activity of lipoprotein lipase and promote chylomicron clearance. In the chronic state, whey proteins affect intestinal lipid absorption, excretion, and de novo cholesterol biogenesis in hepatocytes [259]. Peptides from dairy milk casein offer cholesterol-lowering activity by diminishing cholesterol micellar solubility and absorption and by reducing mRNA expression of acetyl-CoA acetyltransferase-2 and microsomal triacylglycerols (MTP) in proximal intestinal cells [227].

Dairy milk protein-derived peptides may inhibit hypercholesteremic enzymes and associated genes. They inhibit the activities of cholesterol esterase and pancreatic lipase [285] and expression of the lipogenesis-related genes SCD-1, ACC1, and FASN. They increase the peroxisome proliferator-activated receptor-α (PPAR-α) signaling pathway and expression of the β-oxidation-related genes CPT-1a, PPARα, and ACOX1 [286]. Dairy milk casein-derived peptides enhance trans-intestinal cholesterol excretion (TICE) via regulation of the liver X receptor-α (LXR-α) signaling pathway and upregulation of ABCG5. Lys in dairy milk proteins is known to be involved in the lipid accumulation metabolism of long-chain fatty acids, which are crucial for the endogenous synthesis of carnitine. It was found that Lys-supplemented formula reduces the concentration of TGs in tissues [287]. Moreover, the concentration of triacylglycerols and gene expression of lipogenesis enzymes in hepatocytes are suppressed by Leu and Ala in milk proteins and, consequently, are linked with reduced body fat accumulation [288]. Dairy milk protein-derived peptides induce fibroblast growth factor 15/19 (FGF15/19) from enterocytes, which suppresses hepatic bile acid synthesis involved in adjusting hepatobiliary cholesterol [289].

Metabolic diseases, including obesity, are associated with a proinflammatory state and intracellular redox imbalance and the formation of ROS, which result in mitochondrial dysfunction, lipid oxidation, ROS-related impairment, and many other issues [290]. Milk protein-derived peptides offer lipid peroxidation activity due to the presence of Leu or Val and other hydrophobic amino acids, such as Trp, Tyr, His, and Pro. It has been proven that peptides derived from milk proteins can protect Caco-2 cells from lipid peroxidation (peroxide-induced oxidative stress) by modulating the Keap1-Nrf2 pathway [291], which may be responsible for the suppression of excessive body weight [268]. Peptides from milk proteins inhibit the activity of XO, supposed to be the source of ROS that causes atherosclerosis and cholesterol crystals [292].

### 4.3. Biochemical Mechanisms Considering IPP and VPP as Model Peptides

**Figure 7:**
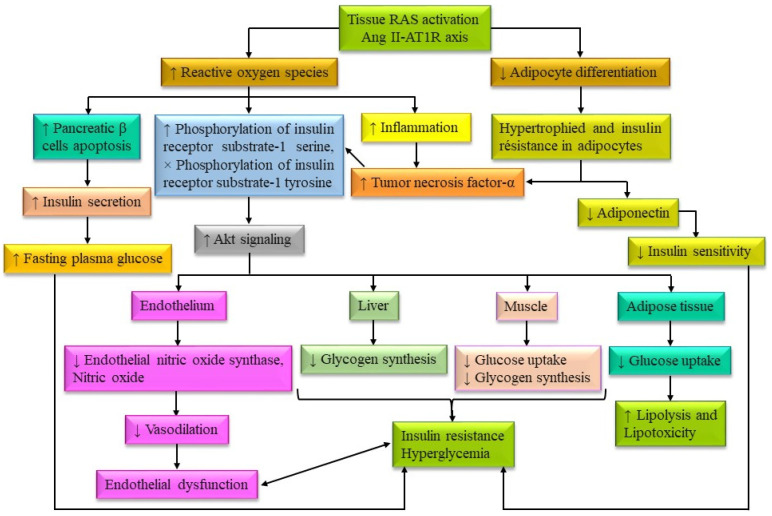
Interconnections between inflammation, oxidative stress, and dysregulation of glucose and lipid metabolism (self-developed; concept was adopted from Putnam et al., 2012 [293]).

Two milk protein-derived tripeptides, IPP and VPP, were meticulously studied concerning their effects on biomarkers of metabolic diseases. Hyperglycemia, hyperinsulinemia, and lipotoxicity are influenced by the expression of local RAS components, especially the Ang II-AT1R axis. Ang II impedes adipocyte differentiation, which promotes adipocyte dysfunction and reduces insulin sensitivity through reduced adiponectin and greater proinflammatory adipokine secretion. Ang II further enhances ROS formation and modulates a wide range of metabolic pathways in different tissues. Inflammation in cells, distressed insulin signaling in the endothelium, hepatocytes, muscle, and adipose tissues promote endothelial dysfunction and insulin resistance. Furthermore, stress-responsive damage to pancreatic β-cells is the cause of lower insulin secretion, which enhances fasting blood glucose [293,294]. It may be realized that understanding the pipelines among inflammation, insulin secretion and resistance, and dysregulation of lipid metabolism will be useful to explain how dairy protein-derived peptides IPP and VPP modulate different biochemical pathways and metabolic biomarkers associated with hyperglycemia and dyslipidemia. In Figure 7, the interconnections between RAS-mediated inflammation, oxidative stress, insulin secretion and resistance, and dysregulation of glucose and lipid metabolism are presented.

Formation of stress-responsive inflammatory cytokines is influenced by several physiological and metabolic factors, such as the activity of the RAS, dysregulation of NADPH oxidase, adipokines, extracellular signal-regulated kinase (ERK), formation of plasminogen activator inhibitor-1 (PAI-1) and C-reactive protein, and activation of the NF-κB pathway, among others [294]. Antioxidant peptides, either direct scavengers or inhibitors of the inflammatory pathways mentioned above, participate in counter-regulatory pathways and act as anti-maladaptation agents in metabolic diseases [295,296]. The ACE inhibitory activity of dairy milk protein-derived IPP and VPP was proven by in vitro studies [297,298], animal models [299,300,301,302], and clinical trials [303,304,305,306]. Note that the effects were not equivocal regarding their biological efficacy [307,308], but there is a consensus that they are ACE inhibitors. ACE prefers to bind peptides containing a hydrophobic (aromatic or branched side-chain) amino acid residue, such as proline, at the C-terminal position [309]. These hydrophobic residues provide the structural basis for the ACE inhibitory effect; however, the downstream effects are not uniform. While both IPP and VPP inhibited the NF-κB pathway, only VPP antagonized the activation of extracellular signal-regulated kinase (ERK) 1/2 in a rat aortic vascular smooth muscle cell (VSMC) line, A7r5 [310]. It was found that administration of VPP and IPP might be beneficial for preventing atherosclerosis due to the activity of ACE and hypercholesterolemia. Plasma lipid levels and 8-isoprostane, a biomarker of oxidative stress, remained unchanged after 31 weeks of oral feeding of IPP and VPP to mice; however, mRNA expression of inflammatory cytokines, such as IL-6 and IL-1β, oxidized low-density lipoprotein receptor, and transcription regulators were reduced [311]. He antihypertensive and cholesterol-lowering effects of the tripeptides IPP and VPP were proven by a 10-week clinical trial. It was reported that systolic (but not diastolic) blood pressure was significantly decreased. Furthermore, total cholesterol and LDL-C were decreased; however, HDL-C, triacylglycerols, and CRP remained unchanged [312]. Bovine casein-derived tripeptides IPP and VPP from casein hydrolyzate have been reported to prevent obesity-induced chronic adipose inflammation in both animal models and cell cultures. High-fat diet-induced systemic inflammatory factors, hypertrophic white adipocytes, and macrophage infiltration were suppressed in C57BL/6J mice due to casein hydrolyzate feeding for 6 weeks. Casein hydrolyzate was able to mitigate adipocyte dysfunction induced by TNF-α by increasing the expression of CCAAT/enhancer binding protein α (C/EBP-α) rather than PPAR-γ in the 3T3-L1 cell line. Furthermore, casein hydrolyzate suppressed mitogen-activated protein kinase (MAPK)-c-JNK phosphorylation and enhanced phosphorylation of ERK 1/2 in TNF-α-induced 3T3-L1 cells [313]. VPP was tested in high-fat diet-fed C57BL/6J mice for 10 weeks to understand its effect on inflammation in adipose tissues. The results showed that the expression level of CD18 in peripheral blood monocytes was significantly decreased compared with the placebo group. This was particularly prominent in the stromal vascular fractions of the fatty tissue of mice treated with VPP. Moreover, the expression of monocyte chemoattractant protein-1 and IL-6 in adipose tissue was also lower [314]. IPP and VPP were able to promote adipocyte differentiation and inhibit inflammation in the murine preadipocyte cell line 3T3-F442A. The upregulation of PPAR-γ and secretion of the protective lipid hormone adiponectin from these cells were reported as underlying mechanisms for beneficial adipogenic differentiation. IPP and VPP inhibited cytokine-induced changes, such as reduction in adipokine levels and activation of the NF-κB pathway [315]. These findings were supported by another study, suggesting that both IPP and VPP can prevent the activity of the inflammatory mediator NF-κB under TNF stimulation in similar cell lines. Under TNF stimulation, they were able to prevent insulin resistance and enhance the expression of GLUT4 [234]. Furthermore, they can enhance glucose consumption in HepG2 cell lines via upregulated protein expression of p-AKT and GLUT2 and regulate the activities of glucose-metabolizing enzymes in HepG2 cells. IPP also directly interacts with the insulin receptor, activating the insulin/AKT signaling pathway; whereas the contribution of VPP to glucose consumption is not attributed to insulin receptor binding [316]. On the other hand, both peptides promoted glucose uptake via the adenosine monophosphate-activated protein kinase (AMPK) pathway, accompanied by GLUT4 translocation rather than the insulin signaling pathway, in the L6 rat myoblast cell line [317]. Therefore, it may be realized that both IPP and VPP can modulate blood glucose levels and may have an impact on glucose-mediated metabolic disorders.

### 4.4. Clinical Investigations

Several acute (less than 1 week) and some long-term (4 to 12 weeks) intervention studies were performed with obese and diabetic individuals to understand the influence of dairy proteins on gastric emptying, gastric secretion, amino acid absorption, and enterogastrone response on serum glucose and insulin levels because they are directly related to diabetic conditions; however, the underlying pipelines are not fully comprehended [191]. Furthermore, results of some clinical investigations proved that dyslipidemia in obese or non-obese subjects is associated with insulin resistance and diabetic conditions. Summarizing all the findings, it may be supposed that dairy milk proteins and peptides derived from them can reduce the risks of hyperglycemic and dyslipidemic incidences by stimulating insulin and secretion of incretin hormones (GLP-1, GIP), suppressing appetite, slowing gastric emptying, and modulating glucose and lipid metabolism. It is noteworthy to mention that co-ingestion of a protein hydrolysate or specific amino acids with intact proteins modulates glucose and lipid metabolism [191,318]. There were no experiments performed specifically for fast food-induced obesity, except [319]. Placebo food formulas associated with modulation of biomarkers of hyperglycemia and dyslipidemia may explain fast food-induced obesity. Therefore, it may be realized that results of some clinical investigations about the impacts of milk proteins and peptides on biomarkers of hyperglycemia and dyslipidemia can explain their role in fast food-induced obesity. Results of some clinical investigations about the effects of milk proteins and peptides on outcomes and biomarkers of hyperglycemia and dyslipidemia are summarized in Table 6.

#### 4.4.1. Dairy Proteins in Meals and T2DM Subjects

In a clinical investigation, it was found that the glucose response was lower when whey protein was included in breakfast and lunch (test meals) for T2DM subjects. The postprandial plasma GIP response was higher in meals with whey; whereas no differences were found in GLP-1 between the reference (ham and lactose) and test meals [320]. In another investigation, it was found that although gastric emptying and plasma insulin, glucagon, C-peptide, GIP, and GLP-1 were improved by the consumption of bovine casein or whey protein with carbohydrates (co-administration) compared with carbohydrate feeding in healthy and prediabetic adults, their effects were similar. Likewise, venous blood glucose was reduced by the two types of protein diets compared with the carbohydrate diet [340]. Interestingly, it was found that even though whey protein can reduce postprandial TG, FFA, and glucose responses more than casein, cod, and gluten protein in a supplemented fat-rich meal without eliciting plasma insulin, glucagon, GLP-1, and GIP in individuals with T2DM, the retinyl palmitate response in the chylomicron-rich fraction was lower; whereas the retinyl palmitate response in the chylomicron-poor fraction was higher with whey protein than with casein protein and cod protein in the fat-rich meal. A higher retinyl palmitate response in the chylomicron-rich fraction compared to the chylomicron-poor fraction signifies more lipolysis rather than lower hepatic clearance [327]. In another investigation, it was found that casein in a fat meal and a carbohydrate-fat meal increased concentrations of plasma insulin and glucagon more than a fat meal in subjects with T2DM. Casein in a carbohydrate-fat meal increased the plasma GIP response; however, no GLP-1 response was noted. The plasma postprandial glucose concentration was also reduced by the fat meal and casein + fat meal compared to the fat-carbohydrate meal. An increase in chylomicron-rich retinyl palmitate compared to chylomicron-poor retinyl palmitate after the casein + fat meal was noted; however, no significant differences were found in postprandial TG levels between the casein-fat and casein-free meals [330].

#### 4.4.2. Dairy Proteins in Preload Formulas and T2DM Subjects

Several investigations indicate that preloading whey protein plays a significant role in carbohydrate-rich meal consumption and physiological responses in subsequent periods. Consumption of whey protein before a carbohydrate meal suppressed gastric emptying and postprandial glycemia; whereas the secretion of plasma insulin, GIP, and CCK was stimulated in subjects with T2DM [326]. It was found that plasma glucose was reduced and insulin and C-peptide responses and GLP-1 were increased in T2DM subjects due to whey preloading of a high-calorie breakfast [338]. In another investigation with T2DM subjects, the overall postprandial iAUC for plasma glucose, ghrelin, and hunger scores were reduced, and greater postprandial overall AUCs for plasma insulin, C-peptide, GLP-1, and satiety scores were noted for a whey protein-based breakfast than for a high-carbohydrate breakfast [342]. Similar results were also reported by other investigators when T2DM subjects consumed WPI before breakfast [339] and both breakfast and dinner [343].

Likewise, whey protein preloading influences the consumption of fat-rich meals and physiological responses after meal consumption. For example, consumption of whey protein before a fat-rich meal reduced gastric emptying and enhanced plasma insulin, glucagon, and GIP responses but did not influence lipid or glucose responses in subjects with T2DM [347]. In another investigation, pronounced responses of plasma insulin and GLP-1 and lower responses of plasma glucose were noted due to WPI consumption prior to a fat-rich meal; however, responses of GIP, FFA, and appetite were not influenced [348].

Furthermore, a reduction in fasting glucose and insulin resistance due to the consumption of a whey protein beverage for 10 weeks in subjects with T2DM before and after 45 early morning high-intensity mixed-mode interval training (MMIT) sessions was reported [344]. Interestingly, it was found that consumption of WPI and lactoferrin with Cys (non-BCAA) prior to breakfast was more potent than casein in reducing insulin resistance and fasting plasma glucose in T2DM subjects. Positive effects also manifested in blood lipid profiles. Total serum cholesterol, TG, and LDL-C levels were reduced by WPI and lactoferrin with Cys. Furthermore, it was found that daily supplementation of undenatured Cys-rich WPI for 3 months, even at a low dose (4625 g/day), improved antioxidative biomarkers (superoxide dismutase, glutathione peroxidase, glutathione, and reduced glutathione-to-oxidized glutathione ratio) and suppressed inflammatory biomarkers (high-sensitivity C-reactive protein, IL-6, tumor necrosis factor-α, and malondialdehyde), which play a central role in diabetes complications and risks of other metabolic diseases [352].

#### 4.4.3. Dairy Proteins in Meals and Obese/Overweight Subjects

Positive effects of whey protein on overweight and obese individuals were also noted because it may be supposed that whey proteins reduce insulin resistance and improve fasting insulin levels. WPI was recognized as a better dietary protein than cod, casein, and gluten protein in a high-fat, high-carbohydrate diet [334] and a vegetable protein-supplemented high-fat diet [350] for obese individuals. It was found that there was a significant decrease in postprandial serum TG after consumption of a whey meal compared to a casein meal; however, postprandial total cholesterol, LDL-C, HDL-C, NEFAs, apolipoprotein B-48, plasma insulin, and leptin were unchanged due to the consumption of casein and whey proteins in obese subjects [329]. In another investigation, it was found that serum total cholesterol, fasting TG, and LDL-C were decreased more by whey protein than by casein protein diets when obese subjects consumed them before breakfast and evening meals. Surprisingly, it was found that fasting plasma glucose was improved after the consumption of whey and casein meals [259]. Similar results were reported by other investigators. It was proven that insulin resistance, serum total cholesterol, adiponectin, LDL-C, HDL-C, and leptin were not changed; however, fasting plasma glucose was increased by both casein and whey protein diet compositions [328]. Furthermore, increases in postprandial GLP-1, insulin, and glucagon and reductions in postprandial GIP, total cholesterol, LDL-C, NEFA, and TG by WPI in a ketogenic diet compared with animal- and plant-based proteins in a high-fat diet were proven [334,350]. Likewise, plasma insulin and total AAs were increased when milk or milk protein was consumed with a high-fat meal by obese individuals; however, serum TG was increased by milk protein [336]. There were no significant changes in fasting plasma insulin, glucose, total cholesterol, LDL-C, whole-body mass, lean mass, fat mass, or thigh muscle area with 1.5 g total protein (MPI)/kg body weight/day compared with 0.8 g total protein (MPI)/kg body weight/day, except for fasting serum TG. The reduction in fasting serum TG was more significant with higher MPI consumption than with lower consumption [319]. Low-fat, high-casein, or high-whey protein diets were considered more effective for weight control than low-fat, high-carbohydrate diets for obese subjects [328].

#### 4.4.4. Dairy Proteins in Preload Formulas and Obese/Overweight Subjects

Positive effects of dairy protein consumption before meals were proven in overweight subjects. In an investigation, it was found that acute appetite and energy intake were equally reduced after consumption of casein- or whey-based preloads compared to glucose-based preloads. This might be due to higher CCK and lower ghrelin responses induced by dairy protein preloads. Postprandial plasma glucose was reduced by protein (mean value of casein and WPI)-based preloads compared to lactose- and glucose-based preloads; however, insulin levels did not change among the different preloads [322]. The impacts of a whey protein meal prior to a fat-rich meal on responses of serum TG and apolipoprotein B-48 in subjects with and without T2DM were investigated. Subsequently, efforts were made to understand whether a whey protein pre-meal had a more pronounced effect on lipid responses than whey protein being part of the fat-rich meal. It was found that a whey protein pre-meal before a fat-rich meal improved plasma insulin, glucagon, and GIP responses in subjects with and without T2DM; however, gastric emptying was delayed by the whey protein pre-meal. Plasma insulin, glucagon, and GIP responses were improved in subjects with and without T2DM by the pre-meal compared to whey protein in the fat-rich meal. Interestingly, it was found that gastric emptying was delayed by the whey protein pre-meal compared to whey protein in the fat-rich meal; however, postprandial serum TG, apolipoprotein B-48, and NEFA levels in subjects with and without T2DM were not significantly changed [347]. In another investigation, it was found that a pre-meal with 20 g of whey protein prior to a high-fat meal stimulated the secretion of insulin and glucagon and reduced blood glucose and gastric emptying compared to a pre-meal with 10 g of whey protein and placebo (no whey protein). There were no changes in GIP, TG, apolipoprotein B-48, or FFA levels with the whey protein pre-meal [348].

An interesting investigation was performed by Danish research groups. The interaction between protein and fat in preload diets was examined to understand all gene expressions and postprandial apolipoprotein B-48, GLP-1, GIP, insulin, glucagon, glucose, triacylglycerol, and FFA levels in obese subjects. The fasting gene expressions of lipoprotein lipase, G protein-coupled receptor 120, and CD36 were upregulated; whereas postprandial gene expression of G protein-coupled receptor 120 and CD36 were downregulated after casein with a high amount of medium-chain saturated fatty acids in the preload diet. No postprandial changes in gene expression were observed with casein and a low amount of medium-chain saturated fatty acids in the preload diet. Upregulation of postprandial lipoprotein lipase gene expression was noted after whey with a high amount of medium-chain saturated fatty acids in the preload diet. The fasting gene expressions of G protein-coupled receptor 120 and CD36 were upregulated by whey with a low amount of medium-chain saturated fatty acids in the preload diet; whereas downregulation of postprandial lipoprotein lipase and G protein-coupled receptor 120 gene expression was noted. Interestingly, no modulation of FABP4 or FAS genes in either fasting or postprandial conditions was noted with any of the proteins and medium-chain saturated fatty acid combinations in the preload diets [341].

#### 4.4.5. Dairy Protein Hydrolysates and T2DM Subjects

The insulinotropic effect of WPH was proven in several investigations, and in many cases, the addition of EAAs offered better results. Some results are mentioned herein. Higher glucose, insulin, glucagon, and GLP-1 responses were contributed by LAPRODAN-DI-3065 (WPH) than by LACPRODAN CGMP-10 (caseinoglycomacropeptide), LACPRODAN-ALPHA-10 (α-lactalbumin), and LAC-PRODAN-DI-9224 (whey protein isolate) in fat-carbohydrate meals for T2DM subjects. However, no significant differences were noted for postprandial TG, retinyl palmitate in the chylomicron-poor fraction, and FFA with any of the dietary proteins; higher levels of retinyl palmitate in the chylomicron-rich fraction were contributed by LACPRODAN-DI-3065 than by all other dietary proteins [333]. It was found that plasma insulin concentration was increased significantly by WPH in a dose-dependent manner, which suppressed the plasma glucose level to the normal range at 2 h after the meal [335]. Likewise, the postprandial plasma glucose response in T2DM subjects was decreased by both whey protein and WPH after breakfast and a mixed-macronutrient meal. Similarly, insulin response and satiety were increased by both intact whey protein and WPH after breakfast and lunch [346]. Furthermore, insulin resistance and postprandial plasma glucose were reduced when obese and type 2 (pre-)diabetic subjects consumed whey protein enriched with Leu and vitamin D [351]. Beneficial outcomes from casein hydrolysate were also reported. It was found that intake of 12 g of casein hydrolysate had a positive effect on post-challenge insulin and glucose levels in subjects with T2DM [332]. The insulin response was increased and the glucose response was decreased by casein hydrolysate compared to carbohydrate diet and intact casein [337,345]; however, no significant difference in gastric emptying outcomes was noted between intact casein and hydrolysate [345]. The effects of different concentrations of milk protein hydrolysates containing a bioactive arginine-proline dipeptide with α-glucosidase-inhibiting properties were investigated in prediabetic subjects. Although the iAUC of plasma glucose was significantly reduced by low-dose milk protein hydrolysates compared to placebo, only a minor insulinotropic effect was noted [349].

#### 4.4.6. Co-Consumption of Amino Acids with Dairy Proteins and T2DM Subjects

Furthermore, it was proven that the addition of specific amino acids to protein formulas markedly promoted the secretion of insulin, leading to a concomitant reduction in the post-challenge plasma glucose response. For example, implementation of Leu and casein hydrolysate with a carbohydrate-based diet offered a greater insulin response, reduced postprandial plasma glucose, and improved blood glucose homeostasis in both T2DM [323,324] and healthy [324] subjects. Similar outcomes were reported in other investigations [321,325,331]. The plasma insulin response was higher with a carbohydrate and protein (50% as casein hydrolysate, 25% as free Leu, and 25% as free Phe) diet than with a carbohydrate diet, and concomitant plasma glucose responses were lower with the carbohydrate and protein diet in T2DM subjects [321]. Consumption of insuVida™, an enzymatic hydrolysate of casein with or without the addition of Leu, could significantly lower plasma glucose compared to placebo and intact casein in T2DM subjects due to its insulinotropic effect [331]. In another investigation with non-obese T2DM subjects, concentrations of postprandial plasma glucose were reduced, and total amino acids, BCAAs, EAAs, insulin, C-peptide, and proinsulin were elicited more by whey protein and/or FAA meals than by casein meals, except for GLP-1 [325].

#### 4.4.7. Contradictory Results

Contradictory results were also reported, mentioned herein. Although it was proven by several investigations that predominant insulin responses and suppression of concomitant plasma glucose responses occur due to co-ingestion of a protein hydrolysate with a meal or prior to a meal, unfortunately, in one experiment, it was found that casein hydrolysate with every main meal did not improve glucose homeostasis over a 24 h period in long-standing T2DM [353]. Smaller postprandial suppression of NEFAs after consumption of LACPRODAN-DI-3065 meals compared with other proteins (LACPRODAN-ALPHA-10, LACPRODAN-DI-9224, and LACPRODAN CGMP-10) in fat-carbohydrate meals was noted in obese non-diabetic individuals; however, no significant differences in body weight or fasting concentrations of plasma glucose, glucagon, CCK, ghrelin, GLP-1, and GIP were noted [354]. Although it was proven that dairy milk proteins are superior to many animal (cod, meat, fish, and egg) [327,334,350] and vegetable (gluten, soya, green pea, and cereal) [342,350] proteins in many investigations, lupin protein compared to casein was more potent in improving the LDL-C:HDL-C ratio in obese/hypercholesterolemic subjects. No significant differences in plasma glucose, Arg, Asn, Cys, Gln, Glu, His, Ile, Leu, Phe, Ser, Trp, or Tyr levels were noted when obese subjects consumed casein and lupin protein, except for plasma TG levels. Plasma TG levels and Ala and Gly were reduced in obese subjects due to the consumption of casein [355]. In another investigation, it was found that there were no differences in postprandial and 24 h energy expenditure or appetite regulation when overweight and moderately obese subjects consumed high concentrations of hydrolyzed casein, intact casein, and intact whey. The results were explained by similar absorption rates when proteins were served as high-protein mixed meals. Lipid oxidation and NEFA concentrations were found to be higher after consumption of intact whey than after consumption of intact and hydrolyzed casein during the daytime and after the breakfast meal [356]. Fasting blood glucose and insulin resistance were significantly increased due to 3 months of consumption of bread fortified with 20 g WPC in overweight/obese subjects with T2DM, and no significant difference compared with placebo was noted. Similarly, consumption of the 20 g WPC-fortified bread led to no significant difference in serum lipid (TG, HDL-C, LDL-C) profile compared with the placebo diet. Investigators realized that 20 g of WPC in bread formulation was not sufficient for providing benefits to overweight T2DM subjects [357]. Other investigators also published similar results. For example, 1.5 g total protein (MPI)/kg body weight/day with an unhealthy Western-style eating pattern did not change fasting plasma insulin, glucose, total cholesterol, or LDL-C levels in obese subjects [319]. In older overweight/obese adults with T2DM, regular consumption of whey protein with vitamin D supplementation for 24 weeks did not offer additional benefits to PRT on measures of glycemic control, body composition, muscle strength, or cardiometabolic risk factors. Investigators felt that there might have been an inadequate concentration of whey protein (20 g whey protein each morning on non-training days with an additional 20 g whey protein after exercise on training days) in the diet, which was insufficient to provide additional PRT benefits in adults with T2DM [170].

#### 4.4.8. Limitations of Clinical Investigations

It is necessary to indicate some limitations of the investigations mentioned in Table 6. While the effects of dairy milk proteins on plasma GLP-1 and GIP were investigated in several studies, very little information was published regarding the effects of milk proteins on the physiological roles of ghrelin [322,342,354], CCK [322,326,354], and DPP-4 [338] in subjects with T2DM. Although dairy protein hydrolysates were used in different investigations [321,323,324,325,331,332,333,335,337,345,346,349,351], the degree of hydrolysis (DH) and reaction conditions of hydrolysis were not reported, except in [335]. DH of protein is an important issue in protein-based dietary formulas because the absorption of lower-molecular-weight peptide and AAs in the bloodstream and biofunctional activity depend on peptide length and amino acid sequence. Furthermore, AA sequences of peptides in protein hydrolysates were not mentioned by investigators, except in [349]. Different criteria for the consideration of obesity, hyperglycemia, and dyslipidemia were mentioned by different authorized committees (WHO, IDF, EGIR, and AACE) [6]. These criteria were considered to describe obesity, hyperglycemia, and dyslipidemia in different investigations, as mentioned in Table 6. Therefore, results from a particular research group were not directly comparable with those from other investigators.

## 5. Conclusions

Metabolic diseases, including obesity, are the consequence of a complex interplay between genetic, dietary, gut microbiota, and environmental factors, and they currently stretch across developed and developing countries. One of the major reasons for obesity is an imbalance of energy intake and expenditure, where high calorie fast foods may have a significant contribution. It is associated with a wide range of physiological factors, such as insulin resistance, hyperglycemia, hypertriglyceridemia, hypoxia, oxidative stress, mitochondrial dysfunction, imbalanced glucose and lipid metabolism, release of adipokines/cytokines/exosomes, angiogenesis, and EMT. The underpinnings of the pathogenesis of obesity are not yet fully understood; however, the contributions of hyperglycemia and dyslipidemia in the case of obesity are recognized. Therefore, obesity is considered one of the key drivers of increased healthcare expenditure. Realizing the contribution of high-calorie fast foods to the prevalence of obesity, efforts have been focused on the modulation of dietary perception and components in the context of obesity management corroborated by hyperglycemia and dyslipidemia.

Dairy proteins emerge as pivotal players in the functional food sector, offering biotherapeutic activities that modulate obesity prevalence. They offer positive effects on postprandial and post-exercise glucose, lipid, and protein metabolism, and may improve metabolic health by reducing body weight and fat mass through enhanced satiety, maintaining the anabolic sensitivity of muscle to nutrition, muscle protein synthesis, and skeletal muscle metabolic function. Diets with higher amounts of dairy protein improve thermogenesis, which influences the balance of dietary calorie consumption and improves energy expenditure. Several epidemiological and cohort studies have confirmed that the consumption of dairy products decreases the prevalence of MS, while experimental studies reveal that dairy proteins and peptides derived from them play a promising role in the modulation of different risk factors of MS. The significance of BCAAs in dairy proteins for the modulation of glucose and lipid metabolism has been recognized. In the present review, possible molecular interactions and biochemical mechanisms related to fast food-induced obesity are mentioned comprehensively. Furthermore, the role of dairy proteins and peptides produced from cow and buffalo milk proteins in the amelioration of fast food-induced obesity has been represented.

Chemical, enzymatic, and recombinant DNA technology have been employed for producing peptides from milk proteins according to the literature survey; however, their production is limited to certain manufacturers, as mentioned in Table 5. This might be due to complicated processing and a lack of awareness of health-promoting benefits and economic factors. The characteristics of dairy protein hydrolysates (FAAs, length, and AA sequence in peptides) are not mentioned by most manufacturers; however, this is an important aspect in protein-based dietary formulations because physiological roles depend on peptide length and amino acid sequence. Therefore, sequencing of peptides produced from dairy milk proteins by liquid chromatography-electrospray ionization quadrupole time-of-flight mass spectrometry (LC-ESI-Q-TOF-MS) might be important for understanding their physiological roles and bio-efficacy comprehensively. Although a lot of scholarly information exists on the synthesis of various milk protein-derived peptides through enzymatic and chemical routes along with their physicochemical information via in silico QSAR approaches, their applications in the food and clinical sectors are hindered to some extent. Therefore, future research efforts should be directed toward evaluating the molecular mechanisms of action and overall possible health-promoting effects in vivo. Production of targeting peptides on an industrial and semi-industrial scale for the development of functional foods could be interesting. Separation of these peptides followed by their encapsulation in order to increase their shelf life and controlled release in the intestinal system would make them astonishing biotherapeutics. Furthermore, masking of the mentioned therapeutic peptides by sweeteners to reduce bittering effect may be investigated by stopped-flow fluorescence, molecular docking, electronic tongue, and clinical trials. The mode of administration (with or without water), time (fed or fasted phase), and inter-individual variability due to sex, age, pathological condition, and ethnicity affect the bioavailability and functional activities of peptides. Therefore, pharmacokinetic information is necessary to understand the effective mode, time, and dosage of administration.

Milk protein hydrolysates have great market value, and their demand is increasing with the progress of time. Presently, some food industries are focusing on marketing individual dairy proteins and peptides along with regular dairy foods; however, this is limited. Protein hydrolysates can be produced from whey, a byproduct of cheese processing. Whey cannot be disposed of directly to the aquatic system due to stricter environmental legislation. It may be supposed that besides the commercialization of regular dairy foods, the production of peptides or protein hydrolysates from whey proteins may bring an economic boon to dairy and biopharmaceutical industries and will be considered a hallmark in the context of zero-waste disposal.

It may be believed that dairy milk protein-derived peptides may be accepted as potential candidates for the treatment of obesity and other metabolic diseases after rectifying the limitations. The multifaceted discussion in this review may open avenues for future investigations and pave the way for impactful therapeutic interventions for the management of fast food-induced obesity.

## Figures and Tables

**Table 1:** Diagnostic criteria proposed for MS (self-developed, concept was adopted from Jha et al., 2023 [6]).

	WHO	IDF	EGIR	AACE
Insulin resistance	Impaired glucose tolerance, Impaired fasting glucose, T2DM, or Lowered insulin sensitivity * plus any 2 of the following.	-	Plasma insulin >75th percentile plus any 2 of the following	Impaired fasting glucose or Impaired fasting glucose plus any of the following based on the clinical judgment
Blood glucose	Impaired glucose tolerance, Impaired fasting glucose, or T2DM	≥100 mg·dL^−1^ (includes diabetes) **	Impaired glucose tolerance or Impaired fasting glucose (but not diabetes)	Impaired fasting glucose or Impaired fasting glucose (but not diabetes)
Dyslipidemia	TG ≥ 150 mg·dL^−1^ and/or High density lipoprotein cholesterol < 35 mg·dL^−1^ in men or <39 mg·dL^−1^ in women.	TG ≥ 150 mg·dL^−1^ or on receiving treatment for TG. High density lipoprotein cholesterol < 40 mg·dL^−1^ in men or <50 mg·dL^−1^ in women or on receiving treatment for High density lipoprotein cholesterol.	TG ≥ 150 mg·dL^−1^ and/or High density lipoprotein cholesterol < 39 mg·dL^−1^ in men or women.	TG ≥ 150 mg·dL^−1^ and High density lipoprotein cholesterol < 40 mg·dL^−1^ in men or <50 mg·dL^−1^ in women
Blood pressure	≥140/90 mm Hg	≥130 mm Hg systolic or ≥85 mm Hg diastolic or on receiving treatment for hypertension.	≥140/90 mm Hg or on receiving treatment for hypertension.	≥130/85 mm Hg
Body weight	Men: waist-to-hip ratio >0.90; Women: waist-to-hip ratio >0.85 and/or BMI > 30 kg·m^−2^.	Increased waist circumference (population specific) plus any 2 of the following.	Waist circumference ≥94 cm in men or ≥80 cm in women.	BMI ≥ 25 kg·m^−2^
Others	Microalbuminuria: Urinary excretion rate of >20 mg·min^−1^ or albumin: creatinine ratio of >30 mg·g^−1^.	-	-	Other features of insulin resistance ***

* Insulin sensitivity was considered under hyperinsulinemic euglycemic conditions, glucose uptake below lowest quartile for background population under investigation. ** In 2003, the American Diabetes Association (ADA) changed the criteria for impaired fasting glucose tolerance from >110 mg·dL^−1^ to >100 mg·dL^−1^. *** Includes family history of T2DM, polycystic ovary syndrome, sedentary lifestyle, advancing age, and ethnic groups susceptible to T2DM. Abbreviations: WHO: World Health Organization, IDF: International Diabetes Federation, EGIR: European Group for the study of Insulin Resistance, AACE: American Association of Clinical Endocrinology.

**Table 2:** Protein profile (g·L^−1^) of cow and buffalo milk (self-developed, information were adopted from Roy et al., 2020 [183]).

	Casein Protein (g·L^−1^)				Whey Protein (g·L^−1^)	
	Total Protein for Cow (24.6–28) and Buffalo (32–40) Milk				Total Protein for Cow (5.5–7) and Buffalo (6) Milk	
	α_s1_-Casein	α_s2_-Casein	β-Casein	κ-Casein	β-Lactoglobulin	α-Lactalbumin
Cow	8–10.7	2.8–3.4	8.6–9.3	2.3–3.3	3.2–3.3	1.2–1.3
Buffalo	8.9	5.1	12.6–20.9	4.1–5.4	3.9	1.4

**Table 3:** Characteristics of dairy milk proteins.

Fraction of Protein		Approximate Molecular Weight (kDa) [196,197]	Amino Acids (-) [196,197]	Isoelectric Point (-) [197,198]	Denaturation Temperature (°C) [198,199]	Allergome Name [200]
Casein	α_s1_-casein	23.6	199	4.9–5.0	-	Bos d 9
	α_s2_-casein	25.2	207	5.2–5.4	-	Bos d 10
	β-casein	24	209	5.1–5.4	-	Bos d 11
	κ-casein	19	169	5.4–5.6	-	Bos d 12
Whey	β-lactoglobulin	18.3	162	5.3	71.9	Bos d 5
	α-lactalbumin	14.2	123	4.8	64.3	Bos d 4
	Immunoglobulins (IgG, IgA, IgM)	IgG 150–161, IgA 385–417, IgM 1000	-	IgG 5.5–8.3	72	-
	Bovine serum albumin	66.4	583	4.9–5.1	72–74	Bos d 6
	Lactoferrin	80	703	8.7	72–85	-
	Lactoperoxidase	78	612	9.8	70	-

**Table 4:** Peptides from enzymatic in vitro hydrolysis of cow and buffalo milk proteins, and their biological functions against the risk factors of MS.

Peptide Sequence	Precursor Protein	Source	Hydrolysis Enzymes	Bioactivities	Ref.
FFVAP	α-casein	Cow milk	Trypsin	ACE inhibitory	[214]
KVLPVPQ	β-casein		Proteinase	Anti-hypertensive	[215]
YKVPQL	β-casein		Proteinase	ACE inhibitory	[215]
KVLPVP	β-casein		Carboxypeptidase	ACE inhibitory	[215]
KVLPVP	β-casein		Carboxypeptidase	Antihypertensive	[215]
YGLF	α-lactalbumin		Trypsin, Pepsin	ACE inhibitory	[216]
YLLF	β-lactoglobulin		Trypsin, Pepsin	ACE inhibitory	[216]
KKLGAPSITCVRRAF	Lactoferrin		Pepsin	Anti-inflammatory	[217]
KKLGAPSITCVRRAF	Lactoferrin		Pepsin	Antioxidant	[218]
KKLGAPSITCVRRAF	Lactoferrin		Pepsin	Anticancer	[219]
MAIPPKKNQDK	κ-casein		Chymosin, Trypsin	Antithrombotic	[220]
AVESTVATLEDΣPEVIESPPE, where Σ is Ser(P)	κ-casein		Chymosin	Modulatory of satiety	[221]
TVQVTSTAV, MAIPKKNQDKTEIP	κ-casein		Chymosin, Papain	Anti-obesity	[222]
PGPIPN	β-casein		Trypsin, Chymotrypsin	Immunomodulatory	[220]
YPSYGLNY	κ-casein		Trypsin, Pepsin, Chymotrypsin	Anti-opioid	[220]
YGLF	α-lactalbumin		Trypsin/Pepsin	ACE inhibitory, Anti-opioid	[223]
YLLF	β-lactoglobulin		Trypsin/Pepsin	ACE inhibitory, Anti-opioid	[223]
HIRL	β-lactoglobulin		Trypsin/Pepsin	ACE inhibitory	[223]
RYLGYL, RYLGYLE, YVPFP	α-casein		-	Anticancer	[224]
YPFPGPI, YPFPG	β-casein		-	Anticancer	[224]
FKCRRWQWRMKK, LGAPSITCVRRAF	Lactoferrin		-	Anticancer	[224]
PYPQ, YFYPE, EMPFK, PQSV	Casein		Trypsin, Alcalase	Antioxidant	[225]
YQKFPQYLQY	Casein		Pepsin, Trypsin	Antihypertensive	[226]
LQPE, VAPFPE, TDVEN, VLPVPQ	Milk casein		Neutrase	Cholesterol lowering	[227]
HLPGRG, QNVLPLH, PLMLP, MFE, GPAHCLL, ACGP	Milk casein		Alcalase, Pronase E	Antidiabetic activity (inhibit three diabetic-related enzymes; such as DPP-IV, α-glucosidase and α-amylase)	[228]
RELEELNVPGEIVEΣLΣΣΣEESITRINK	β-casein		Chymotrypsin	Immuno-stimulatory	[229]
LVYPFPGPI	β-casein	Buffalo milk	Proteinase	ACE inhibitory	[230]
FVAPFPE	αs_1_-casein		Trypsin	ACE inhibitory	[231]
YQQPVL	β-casein		Fermentation + Pepsin + Trypsin	ACE inhibitory	[232]
FPGPIPK, IPPK, IVPN, QPPQ	β-casein		Papain, Pepsin, or Trypsin	ACE inhibitory	[233]
IPP/VPP	β-casein		-	Anti-diabetic, Antihypertensive	[205]
IPP/VPP	β-casein		Proteases	Antihypertensive	[234]
RNAVPITPTLNR	αs_2_-casein		Protease	Antidiabetic/α-glucosidase inhibitory	[235]
TKVIPYVRYL	αs2-casein		Protease	Antidiabetic/α-glucosidase inhibitory	[235]
YLGYLEQLLR	αs_2_-casein		Protease	Antidiabetic/α-glucosidase inhibitory	[235]
FALPQYLK	αs_2_-casein		Protease	Antidiabetic/α-glucosidase inhibitory	[235]
YVEELKPTPEGDL	β-lactoglobulin		Pepsin	Antioxidant	[236]
VLPVPQK	β-casein		Pepsin + Trypsin	ACE inhibitory	[237]
VLPVPQK	β-casein		Pepsin + Trypsin	Antioxidant	[238]
YPSG, HPFA, KFQ	β-casein		Papain, Pepsin, Trypsin	Antioxidant	[233]
RELEE, TVA, MEDNKQ	Casein		Trypsin, Alcalase	Antioxidant	[225]

**Table 5:** Commercial peptides from dairy milk proteins (self-developed, information were adopted from Carrasco-Castilla et al., 2012 [249]).

Commercial Brand Name	Food Type	Peptide Sequence	Health/Functional Claim	Manufacturers
Ameal bp^®^, Ameal peptide^®^	Tablets	VPP, IPP derived from β-casein and κ-casein	Reduction in blood pressure	Calpis Co., Tokyo, Japan
BioZate 1	Hydrolyzed whey protein isolate	Fragments from β-lactoglobulin	Reduction in blood pressure	Davisco Foods International Inc., Savage, MN, USA
BiPRO WPI	WPI	GMP (106–109)	Antithrombic and Anticariogenic	Davisco Foods International Inc., Savage, MN, USA
Calpis	Sour milk	IPP and VPP	Hypotensive	Calpis Co., Tokyo, Japan
Calpico^®^/Calpis^®^ AMEALs	Fermented milk	IPP and VPP	Hypotensive	Calpis Co., Tokyo, Japan
C12 peptide	Peptide ingredient	FFVAPFPEVFGK	Hypotensive	DMV International, Veghel, The Netherland
Evolus^®^	Fermented milk	IPP and VPP	Reduction in blood pressure	Valio Ltd., Helsinki, Finland
GC tooth mousse	Water-based creme	Caseinomacropeptide	Anticariogenic	GC Europe N.V., Leuven, Belgium
Glycomacropeptide (GMP)	Fresh cheese, WPI	κ-casein f(106–169)	Anticariogenic and Antithrombotic	Davisco Foods International Inc., Savage, MN, USA
insuVida^TM^	Tablets	Casein hydrolysate	Antidiabetic	DSM Food Specialties, Delft, The Netherland
Lactium^®^	Milk protein hydrolysate	α_s1_-casein f(91–100) YLGYLEQLLR	Reduction in stress effects	Ingredia Nutritional, Arras, France
LACPRODAN-DI-3065	Healthy food formula	Whey protein hydrolysate (WPH)	Benefits to sufferers from impaired digestion as a result of surgery, illness or health conditions, such as short bowel syndrome, pancreatic insufficiency and inflammatory bowel disease.	Arla Foods Ingredients Group P/S, Viby, Denmark
LACPRODAN-DI-3091	Healthy food formula	WPH	Supplements for patients with maldigestion or malabsorption.	Arla Foods Ingredients Group P/S, Viby, Denmark
LACPRODAN-DI-3092	Healthy food formula	WPH	Supplements for patients with maldigestion or malabsorption.	Arla Foods Ingredients Group P/S, Viby, Denmark
LACPRODAN-IF-3070	Healthy food formula	WPH	Sustainable gastrointestinal system for infant	Arla Foods Ingredients Group P/S, Viby, Denmark
LACPRODAN-IF-3071	Healthy food formula	WPH	Sustainable gastrointestinal system for infant	Arla Foods Ingredients Group P/S, Viby, Denmark
LACPRODAN-IF-3080	Healthy infant food formula	WPH	Lower allergenic	Arla Foods Ingredients Group P/S, Viby, Denmark
LACPRODAN-IF-3090	Healthy infant food formula	WPH	Lower allergenic	Arla Foods Ingredients Group P/S, Viby, Denmark
LACPRODAN-DI-2021	Healthy food formula	Casein phosphopeptides	Increase calcium absorption and bone health	Arla Foods Ingredients Group P/S, Viby, Denmark
LACPRODAN-CGMP-10 and CGMP-20	Healthy food formula	Fragment from α-lactalbumin	Improves sleep and memory	Arla Foods Ingredients Group P/S, Viby, Denmark
MI paste^TM^/MI paste plus^TM^	Toothpaste	Caseinomacropeptide	Anticariogenic	GC America, Alsip, IL, USA
Pep2Dia^®^	Capsule (vegetable fiber with 350 mg of milk protein hydrolysate/capsule)	RP	Antidiabetic	Ingredia S.A., Arras Cedex, France
PROTARMOR™ 80	Ingredient	Casein hydrolysates	Weight loss	Armor Proteines, Maen Roch, France
Recaldent^TM^	Ingredient	Caseinomacropeptide	Anticariogenic	Cadbury Enterprises Pte. Ltd., Jurong, Singapore
Trident xtra care^TM^	Chewing gum	Caseinomacropeptide	Anticariogenic	Cadbury Adams, East Hanover, NJ, USA

**Table 6:** Effects of milk proteins and peptides on outcomes and biomarkers of hyperglycemia and dyslipidemia.

Publication Year/Reference	Study Design, Duration	Characteristics and Number of Subjects (n)	Dairy Milk Proteins Formula	Comparison Formula	Effects and Remarks	
					Hyperglycemia	Dyslipidemia
2005/[320]	Randomized, Acute (2 separate occasions with ≥1 week gap between each)	T2DM (14)	Whey protein (27.6 g)	Ham (96 g) and Lactose (5.3 g)	↓ Postprandial plasma glucose by whey protein. ↑ Plasma insulin response and postprandial GIP by whey protein. → GLP-1 by whey protein and ham.	-
2005/[321]	Randomized order and a double-blind (2 trials, separated by a 2 weeks gap between each)	T2DM (10)	No diabetes (n = 10), 0.7 g carbohydrate/kg body weight/h (50% glucose and 50% maltodextrin) with or without 0.35 g/kg body weight/h of a protein hydrolysate and AA mixture (50% casein hydrolysate, 25% free Leu and 25% free phenylalanine) every 15 min until t = 165 min	No diabetes (n = 9), 0.7 g carbohydrate/kg body weight/h (50% glucose and 50% maltodextrin) with or without 0.35 g/kg body weight/h of a protein hydrolysate and AA mixture (50% casein hydrolysate, 25% free Leu and 25% free phenylalanine) every 15 min until t = 165 min	↑ Plasma insulin and ↓ plasma glucose responses by carbohydrate + protein trial than carbohydrate trial in diabetic subjects and matched to control subjects.	-
2006/[322]	Randomized cross-over (4 occasions with 7 days gap between each)	Overweight (19)	Preloads made by water solutions of (a) WPI (55 g), (b) Calcium caseinate (55 g), (c) Glucose (60 g), (d) Lactose (56 g)	-	↓ Acute appetite and energy intake by casein- or whey-, lactose- than glucose-preload. ↓ Postprandial plasma glucose by protein (mean value of casein and WPI)-preload than lactose- and glucose-preload. → Plasma insulin by different preloads. ↑ CCK response and ↓ ghrelin by protein preload.	-
2006/[323]	Randomized, double-blind, placebo-controlled, cross-over, acute challenge (Participants consumed 3 meals/day in a single 24 h period)	T2DM (11), Healthy (11)	T2DM (11), Casein hydrolysate (0.3 g/kg body weight) + Leu (0.1 g/kg body weight) + meal (64% carbohydrate, 25% fat and 11% protein)	Healthy (11), Flavored water + meal (64% carbohydrate, 25% fat and 11% protein)	↓ Plasma glucose by casein hydrolysate in average 24 h by casein and Leu supplemented meal.	-
2006/[324]	Acute, randomized, double-blind (3 trials, separated by at least 1 week gap between each)	T2DM (10), Healthy (10)	T2DM (10), (a) Carbohydrate (50% glucose and 50% maltodextrin) (0.7 g/kg body weight), (b) Carbohydrate + Casein hydrolysate (0.7 g/kg body weight + 0.3 g/kg body weight), (c) Carbohydrate + Casein hydrolysate + Leu (0.7 g/kg body weight + 0.3 g/kg body weight + 0.1 g/kg body weight)	Healthy (10), Similar diet like diabetic group	↑ Insulin response and ↓ plasma glucose response in subjects with T2DM and control subjects by Carbohydrate + Casein hydrolysate and Carbohydrate + Casein hydrolysate + Leu for both T2DM and healthy subjects (Carbohydrate diet basis).	-
2007/[325]	Double-blind, randomized, controlled cross-over clinical trial (5 days study with 2 weeks gap between each)	Non-obese, T2DM (12)	Mixed meal (≈31% carbohydrates, ≈17% lipids and ≈52% proteins as total calories) with (a) WPI (fast protein), (b) micellar casein (slow protein), (c) a mixture of FAAs resembling the AA composition of micellar casein.	-	↓ Postprandial plasma glucose by FAA meal than WPI meal and casein meal. ↑ Plasma BCAAs, EAAs, C-peptide, insulin and pro-insulin concentrations by WPI meal than casein meal and similar with FAA meal. ↓ Plasma GLP-1 response by casein meal than WPI meal. ↑ Plasma GIP response by WPI meal and casein meal than FAA meal.	-
2009/[326]	Acute (3 occasions)	T2DM (8)	(a) Pre-meal: 55 g whey in 350 mL beef soup, (b) Main-meal: 55 g whey in potato	No whey in pre-meal and main meal	↑ Plasma insulin, GIP and CCK by whey diet. ↓ Gastric emptying and postprandial glycemia by whey diet.	-
2009/[327]	Randomized crossover (4 separate occasions with 2–5 weeks gap between each)	T2DM (12)	Whey protein (45 g) in meal (80 g fat and 45 g carbohydrate)	(a) Casein (45 g), (b) Cod (45 g), (c) Gluten (45 g) in meal (80 g fat and 45 g carbohydrate)	↑ Postprandial plasma insulin and incretins (GLP-1, GIP) by whey protein. → Glucagon by all types of proteins.	↑ Postprandial lipemia by whey protein than other proteins.
2009/[328]	Randomized (Energy restriction period of 5–6 weeks followed by a weight maintenance period of 12 weeks with 1 week in-between to change back from liquid to normal food)	Overweight and obese (48)	(a) Casein, (b) Whey protein supplements (2 × 25 g/day)	Maltodextrin	↑ Plasma glucagon. Fasting glucose in normal range by protein diet. → Plasma insulin resistance by casein and whey proteins.	↑ Serum TG by maltodextrin. → Serum total cholesterol, leptin, adiponectin, LDL-C and HDL-C by protein diets.
2010/[329]	Randomized, single blind, three-way crossover design (3 separate intervention days, each preceded by a 1 week washout period)	Overweight or obese (20)	(a) WPI (45 g), (b) Sodium caseinate (45 g)	Glucose (45 g)	↓ Plasma glucose by WPI than sodium caseinate and glucose.	↓ Serum TG-enriched lipoprotein by WPI than casein and glucose. → Serum total cholesterol, LDL-C, HDL-C, NEFA, apolipoprotein B-48, insulin and leptin.
2010/[330]	Randomized, controlled, cross-over, acute challenge trial (4 test meals with intervals of >2 weeks)	T2DM (11)	(a) Casein (45 g) + Carbohydrate (45 g) + Fat (80 g), (b) Casein (45 g) + Fat (80 g), (c) Carbohydrate (45 g) + Fat (80 g)	Fat (80 g)	↑ Plasma insulin and glucagon by Casein + Fat meal and Casein + Carbohydrate + Fat meal than Fat-meal. ↑ GIP response by Casein + Carbohydrate + Fat meal. → GLP-1 response.	→ Serum TG and retinyl palmitate in the chylomicron-rich fraction for all meals. ↑ Retinyl palmitate in the chylomicron-poor fraction by 45 g of protein as casein.
2011/[259]	Randomized parallel (12 weeks)	Overweight and obese (70)	(a) WPI (27 g) in 250 mL of water, (b) Sodium caseinate (27 g) in 250 mL of water. Twice in a day	Glucose (27 g) in 250 mL of water	→ Plasma glucose by WPI than control or casein meal. ↑ Fasting plasma insulin by whey protein.	↓ Fasting serum TG, plasma total cholesterol and LDL-C by WPI.
2011/[331]	Randomized double-blind partial cross-over (3 out of 4 treatments separated by a week gap between each)	T2DM (36)	(a) Intact casein, (b) Casein hydrolysate, (c) Casein hydrolysate with Leu	Carbohydrate (maltodextrin and glucose monohydrate) without (a) intact casein, (b) unhydrolyzed casein, (c) Casein hydrolysate and Leu	↓ Plasma glucose by casein hydrolysate with or without Leu.	-
2011/[332]	Randomized placebo controlled double blind (Separate 3 days with 1 week gap between each)	T2DM (13)	In a single oral bolus (300 mL) containing 50 g of carbohydrates (50% glucose and 50% maltodextrin and casein hydrolysate (6 g or 12 g)	No casein hydrolysate	→ Plasma insulin and glucose by 6 g of casein hydrolysate. ↑ Post-challenge plasma insulin and ↓ glucose levels by 12 g of casein hydrolysate.	-
2012/[333]	Single blind crossover (4 separate occasions with a washout period ⩾2 weeks)	T2DM (12)	Protein supplement ((a) LACPRODAN-ALPHA-10 (45 g), (b) LACPRODAN-DI-9224 (45 g), (c) LACPRODAN CGMP-10 (45 g), (d) LACPRODAN-DI-3065 (45 g)) + Fat (80 g) + Carbohydrate meal (45 g)	-	↑ Plasma glucose, insulin, glucagon and GLP-1 by LACPRODAN-DI-3065 than other proteins in fat-carbohydrate meals.	→ Postprandial serum TG by all dietary proteins. ↑ Retinyl palmitate in the chylomicron-rich fraction by LACPRODAN-DI-3065 than dietary proteins. → FFA and Retinyl palmitate in the chylomicron-poor fraction by all dietary proteins.
2013/[334]	Randomized crossover (4 different meals in different days with a 2 weeks washout period between each meal)	Obese non-diabetic (11)	WPI + Fat (80 g) + Carbohydrate (45 g)	Carbohydrate (45 g) + Fat (80 g) + (a) Cod or casein (45 g), (b) gluten protein (45 g)	↑ Biomarkers (GLP-1, insulin, glucagon) of postprandial glycemia, ↓ Postprandial GIP by WPI. The larger initial plasma insulin and glucagon response after whey meal did not correlate with the initial GLP-1 or GIP responses.	↓ Postprandial serum TG by WPI than cod and gluten proteins. ↓ NEFA by whey and casein than cod and gluten proteins. → Retinyl palmitate in chylomicron by all types of dietary proteins.
2013/[335]	Double-blind, randomized (Each trial was performed on a distinct day with 3 days intervals between each trial)	T2DM (10)	(a) WPH beverage (0.1 g/kg body weight, 0.2 g/kg body weight and 0.4 g/kg body weight), (b) WPI beverage (0.1 g/kg body weight, 0.2 g/kg body weight and 0.4 g/kg body weight)	Distilled water	↑ Postprandial plasma insulin and ↓ post-challenge plasma glucose by 0.2 g/kg body weight WPH or 0.4 g/kg body weight WPI. ↑ Postprandial plasma insulin and ↓ concomitant glucose level to normal range at 2 h after 0.2 g/kg body weight WPH.	-
2013/[336]	Randomized crossover (4 test days, which were separated by a washout period of at least 3 days)	Obese men (16)	Butter cake (high fat) + (a) 500 mL milk or (b) 500 mL water + milk protein 23.4 g or (c) 500 mL water + calcium 2.3 g	Butter cake + 500 mL water	↑ Plasma insulin and ↓ glucose by milk protein. ↑ Plasma concentrations of total AAs, EAAs and non-EAAs by milk and protein milk.	↑ Serum TG by milk protein. ↓ Apolipoprotein B-48 by calcium meal compared with milk. → NEFA by all drinks.
2014/[337]	Double-blind randomized and cross-over (3 trials was separated by at least 6 days gap between each)	T2DM (60)	(a) Carbohydrate + Intact casein (0.7 g/kg body weight + 0.3 g/kg body weight), (b) Carbohydrate + Casein hydrolysate (0.7 g/kg body weight + 0.3 g/kg body weight)	Carbohydrate (50% glucose + 50% maltodextrin) (0.7 g/kg body weight)	↑ Plasma insulin and ↓ plasma glucose responses by Carbohydrate + Protein diet than Carbohydrate diet. ↑ Plasma insulin response by Carbohydrate + Casein hydrolysate diet than Carbohydrate + Intact protein diet. ↓ Plasma glucose response by Carbohydrate + Casein hydrolysate diet than Carbohydrate + Intact protein diet.	-
2014/[338]	Randomized, open-label crossover (2 meals in 2 separate days and a at least 2 weeks gap between each)	T2DM (15)	Preload: 50 g whey in 250 mL water	Preload: 250 mL water	↓ Plasma glucose, ↑ plasma insulin, ↑ C-peptide, ↑ GLP-1, and → DPP4 by whey pre-load.	-
2015/[339]	Randomized, cross-over study (4 weeks)	T2DM (7)	Preload: WPI (25 g) + 25 g chocolate-flavor in 100 mL water	Preload: 25 g chocolate-flavor without WPI in 100 mL water	↓ Postprandial plasma blood glucose and peak blood glucose by WPI formula.	-
2015/[340]	Single-center, randomized, single blind (3 blind challenges on 3 different occasions with a minimum of 2 days gap between them)	Healthy (15), Prediabetic (15)	50 g casein or WPI + 50 g of maltodextrin with dextrose equivalent 19 + 2 g of hydroxyproline + 10 g of lactulose in 300 mL of water.	50 g of maltodextrin with dextrose equivalent 19 + 2 g of hydroxyproline + 10 g of lactulose in 300 mL of water.	↑ Plasma insulin, glucagon, C-peptide, GIP, GLP-1 and satiety by casein and whey proteins than glucose. Plasma glucose by casein and whey proteins than glucose. → All effects two proteins.	-
2015/[341]	Randomized, parallel-controlled, double-blinded (Each test meal with 12 weeks intervention period)	Obese (52)	(a) Whey + Low medium-chain saturated fatty acids, (b) Whey + High medium-chain saturated fatty acids, (c) Casein + Low medium-chain saturated fatty acids, (d) Casein + High medium-chain saturated fatty acids	-	↓ Postprandial apolipoprotein B-48 response after whey compared with casein independently of fatty acid composition. ↑ Postprandial plasma GLP-1 by casein compared with whey. → Postprandial plasma insulin, glucose, glucagon, or GIP among groups.	→ Postprandial serum triacylglycerol and FFA among groups.
2017/[342]	Randomized open-label parallel-arm (12 weeks, dietary intake on 3 days in each week)	T2DM (56)	(a) Whey protein breakfast: 25% fat + 50% carbohydrates + 25% protein (28 g whey from 42 g total protein). (b) Protein breakfast: 25% fat + 50% carbohydrates + 25% (42 g) protein mainly from eggs (7 g), tuna (20 g), soya (7 g).	Carbohydrate breakfast: 25% fat + 64% carbohydrates + 11% (17 g) soya protein	↓ Overall plasma postprandial incremental area under curve (iAUC) for glucose, ghrelin and hunger scores by whey protein breakfast and protein breakfast than carbohydrate breakfast. ↑ Postprandial plasma overall iAUC for insulin, C-peptide, GLP-1 and satiety scores by whey protein breakfast than protein breakfast and carbohydrate breakfast.	-
2017/[343]	(a) Parallel-armed acute challenge (One serving (21 g) of whey protein) and (b) Crossover design (continuous glucose monitoring (CGM) twice, over 2 consecutive weeks, 3.5 days each week)	n = 18 underwent a challenge test (not crossover design). n = 22 underwent CGM and controlled feeding twice (crossover design), Two consecutive weeks: one week WPI and other week placebo diet.	WPI (21 g protein + 3 g carbohydrate + 0.5 g fat)	Indigestible potato starch (1 g protein + 25 g carbohydrate 20 g fiber + 0.5 g fat)	Acute challenge studies: ↑ Plasma insulin, ↑ GLP-1, ↓ Plasma glucose and ↓ Ghrelin by WPI diet. Placebo diet had no effect.	↓ Hypertriglyceridemia by WPI diet.
2018/[344]	Randomized, double-blind, placebo-controlled (10 weeks)	T2DM (24)	WPI beverage (20 g protein + 10 g carbohydrate + 3 g milk fat)	Without WPI (30 g carbohydrate + 3 g milk fat)	↓ Fasting plasma blood glucose and insulin resistance by whey protein diet.	-
2018/[345]	Randomized crossover (2 separate test days with 1 week gap between each)	Overweight and obese (9)	Breakfast (74 g carbohydrate, 2.1 g fat and 8.5 g protein in 100 mL orange juice) with 10% (*w*/*v*) solution of a novel casein hydrolysate	Breakfast (74 g carbohydrate, 2.1 g fat and 8.5 g protein in 100 mL orange juice) with 10% (*w*/*v*) solution of sodium caseinate (intact protein).	→ Gastric emptying outcome by intact casein and hydrolysate. ↑ Insulin response and ↓ glucose response by casein hydrolysate compared to intact casein.	-
2018/[346]	Randomized, single-blind crossover (3 separated occasions separated by 7 days gap between each)	T2DM (11)	(a) Intact whey protein (15 g), (b) WPH (15 g) before mixed-macronutrient breakfast and lunch meals, separated by 3 h.	Flavored water	↓ Plasma glucose by WPH (early) and intact whey protein. ↑ Satiety and Plasma insulin by both WPH and intact whey protein.	-
2018/[347]	Acute, randomized, cross-over (Test days separated by a washout period of approximately 1 week gap between each)	Non-diabetic (12), T2DM (12)	Preload: 20 g whey protein in 200 mL water as pre-meal or part of the fat-rich meal.	Preload: 200 mL water	Plasma ↑ insulin, ↑ glucagon, ↑ GIP and ↓ gastric emptying in subjects with and without T2DM by whey protein pre-meal. Plasma ↑ insulin, ↑ glucagon, ↑ GIP and ↓ gastric emptying in subjects with and without T2DM by pre-meal than main meal.	→ Postprandial TG, apolipoprotein B-48 and NEFA in subjects with and without T2DM.
2018/[348]	Acute, randomized, cross-over (The test days were separated by a wash-out period of approximately 1 week gap between each)	MS (20)	Preload: (a) 10 g whey protein, (b) 20 g whey protein	Preload: No whey protein	↑ Insulin response, ↑ postprandial glucagon, ↓ glucose and ↓ gastric emptying after a pre-meal with 20 g whey protein than 10 g whey protein and placebo. → GIP by whey protein pre-meal.	→ TG, apolipoprotein B-48 and FFA by whey protein pre-meal.
2019/[349]	Randomized, double-blind, placebo-controlled, monocentric, 3-way-cross-over (6 weeks, a wash-out period of 7 days between each study day)	Prediabetic (21)	Milk protein hydrolysate (1.4 g and 2.8 g), 2 dosages/day	Maltodextrin with dextrose equivalent of 9	↓ iAUC of plasma glucose by milk protein hydrolysate in dose-dependent manner. → Insulinotropic properties were insignificant.	-
2020/[350]	Prospective randomized pilot study (45 days)	Obese and insulin resistance (48)	Protein diet (90 g/meal), 5 meals/day for (a) whey protein, (b) vegetable protein (soya, green peas, or cereals), (c) animal protein (meat, fish, egg)	-	↓ Insulin resistance and fasting glycemia by whey protein.	Serum ↓ total cholesterol, LDL-C and TG by whey protein.
2020/[351]	Double-blind randomized clinical trial (13 weeks)	Obese and type 2 (pre-)diabetes (123)	21 g of Leu-enriched whey protein ((3 g total Leu), 9 g carbohydrates, 3 g fat, 800 IU cholecalciferol (Vitamin D3))	Carbohydrate (25 g) and fat (6 g) mix	↓ Insulin resistance and postprandial plasma glucose by Leu-enriched whey protein.	-
2020/[352]	Single-center randomized, placebo-controlled (3 months)	T2DM (120)	Cys-rich (2.7%) WPI with a standardized lactoferrin in 100 mL of water	5 g casein in 100 mL of water	↓ Fasting plasma glucose by Cys-rich WPI diet.	Serum ↓ total cholesterol, triacylglyceride and LDL-C levels by Cys-rich (2.7%) WPI than placebo.
2020/[319]	Randomized, parallel, placebo-controlled, double-blind study (20 weeks: 1 week pre-study measurement period, a 3 weeks baseline period and a 16 weeks energy restriction intervention period	Overweight or obese	n = 21, Unhealthy western-style eating patterns + 1.5 g total protein (MPI)/kg body weight/day	n = 23, Unhealthy western-style eating patterns + 0.8 g total protein (MPI)/kg body weight/day	→ Fasting plasma insulin and glucose	Serum ↓ fasting TG, → total cholesterol and LDL-C

Legend: ↓: decrease, ↑: increase, →: unchanged or insignificant effect, -: not mentioned, +: and.

## Data Availability

Not applicable.
